# Supplementary material for: Elucidating Human Milk Oligosaccharide biosynthetic genes through network-based multi-omics integration
Source: Nat Commun. 2022 May 4;13:2455. doi: 10.1038/s41467-022-29867-4 (PMC9068700; doi:10.1038/s41467-022-29867-4)
Supplement: Supplementary file 1 — Supplementary Information [file 41467_2022_29867_MOESM1_ESM.pdf]

SUPPLEMENT FOR: Elucidating Human Milk Oligosaccharide biosynthetic genes through network-based multi-omics integration

Benjamin P. Kellman[1,3,5,*], Anne Richelle[1,*], Jeong-Yeh Yang[6], Digantkumar Chapla[6], Austin W. T. Chiang[1], Julia A. Najera[1], Chenguang Liang[1,5], Annalee Fürst[1], Bokan Bao[1,3,5], Natalia Koga[1], Mahmoud A. Mohammad[4], Anders Bech Bruntse[1], Morey W. Haymond[4], Kelley W. Moremen[6], Lars Bode[1,7], Nathan E. Lewis[1,5,§]

1. Department of Pediatrics, University of California, San Diego, La Jolla, CA 92093, USA

3. Bioinformatics and Systems Biology Graduate Program, University of California, San Diego, La Jolla, CA 92093, USA

4. Department of Pediatrics, Children's Nutrition Research Center, US Department of Agriculture/Agricultural Research Service, Baylor College of Medicine, Houston, Texas 77030, USA

5. Department of Bioengineering, University of California, San Diego, La Jolla, CA 92093, USA

6. Complex Carbohydrate Research Center, University of Georgia, Athens, GA, USA.

7. Larsson-Rosenquist Foundation Mother-Milk-Infant Center of Research Excellence (MOMI CORE), University of California, San Diego, La Jolla, CA 92093, USA

* These authors contributed equally

[§]Correspondence to: Nathan E. Lewis, nlewisres@ucsd.edu

1. CONTENTS

1 Glossary ... 3

2 Supplementary Figures ... 5

3 Supplementary Datasets and Tables ... 27

4 Supplemental Methods ... 33

4.1 Generation of complete HMO biosynthesis network ... 33

4.2 Reduction of the generic network to a reduced network using Flux Variability approach 33

4.3 Generation of all minimal subnetworks for every potential HMO structure combination . 33

4.4 Assessment of consistency of experimental data with subnetworks and selection of candidate models ... 34

4.4.1 Computation of Subnetwork Score ... 34

4.4.2 Ranking subnetwork performance and extraction of candidate models ... 35

4.4.3 Summary network Extraction from the Reduced Network ... 35

4.5 Resolution of ambiguous genes and HMO structure through analysis of candidate models and subnetwork statistics ... 35

4.5.1 Enrichment of ambiguous HMO structures within top-performing candidate models 36

4.5.2 Enrichment of ambiguous enzymes within top-performing candidate models ... 36

5 Theory, Calculations and Definitions ... 37

5.1 Defining the Model-Space: Input date, Reaction Rules, Complete Network and Reduced Network ... 37

5.2 Identifying Candidate Models within the Reduced Network ... 38

5.3 Defining the Candidate Model Score ... 38

5.4 Flux Balance Analysis (FBA) and Flux Variability Analysis (FVA) ... 39

5.5 Mixed-Integer Linear Programming (MILP) ... 40

6 Supplementary Results ... 41

6.1 Candidate gene filtering by expression and acceptor specificity ... 41

6.2 Generation of 44,984,988 candidate models representing HMO biosynthesis ... 42

6.3 Selection of best performing models ... 42

7 Supplemental Discussion ... 43

7.1 Validity of Selected Transcription Factors ... 43

7.2 Leveraging HMO pathway resolution to clarify natural variation in HMO composition ... 43

7.3 Leveraging HMO pathway resolution to facilitate chemoezymatic synthesis ... 44

8 Supplemental References ... 45

1 GLOSSARY

Term	Definition	Full Definition
Reaction rules, elementary or fundamental reactions	The generic reaction types the can occur in the model	Section 4.1
Candidate structures, isomers	Alternative and proposed structure for HMOs that are not yet fully characterized	**Supplementary Figure** 5
Candidate gene, isoform	All genes in a glycosyltransferase family that could reasonably perform the corresponding reaction.	**Table 1**
Complete Network	The collection of all reactions into a network that will produce all possible glycans up to a pre-specified size	Section 4.1
Reduced Network	A trimming, via FVA (Section 5.4), of to Complete Network to remove reactions unnecessary to reach observed glycans	Section 4.2, 5.4
Candidate Models	Many subnetworks of the Reduced Network, via MILP (Section 5.5), where each subnetwork is capable of uniquely reproducing the observed data	Section 4.3, 5.5
Flux & Normalized flux	Relativistic measure of the stoichiometrically balanced movement of material through a biosynthetic model. Flux can be normalized relative to an upstream reaction to consider substrate limitations. Flux is calculated using Flux Balance Analysis.	**Supplementary Figure** 7<br>Section 5.4
Gene-Linkage Score (GLS)	The highest Spearman correlation between the total normalized flux of through a reaction type and the expression of all corresponding candidate genes. The selection of the "best" genes to describe a linkage within a model.	Section 4.5.2
Model Score	The average gene-linkage score across all 10 fundamental reaction types for a model	Section 4.4.1
High-performing models	Top 5% of candidate models ranked by model score relative to a normal distribution.	Section 4.4.2

Commonly high-performing models	High-performing models using data from both cohorts	Section 4.4.2
Summary Network	A visualization of the most important pathways though the Reduced Network; those most frequently and substantially used in commonly high-performing models	Section 4.4.3
Proportion (PROP)	The hypergeometric enrichment of models where a specific gene was best (highest gene-linkage score within the linkage) in the set of commonly high-performing models vs the background of all models.	Section 4.5.2
Model Contribution Score (MSC)	The Pearson correlation between model score and the gene-linkage score for a particular gene.	Section 4.5.2
Reaction Support Score	The degree to which a gene is likely to “support” a fundamental reaction based on the aggregation of PROP, GLS, and MSC across independent datasets.	Section 4.5.2

2 SUPPLEMENTARY FIGURES

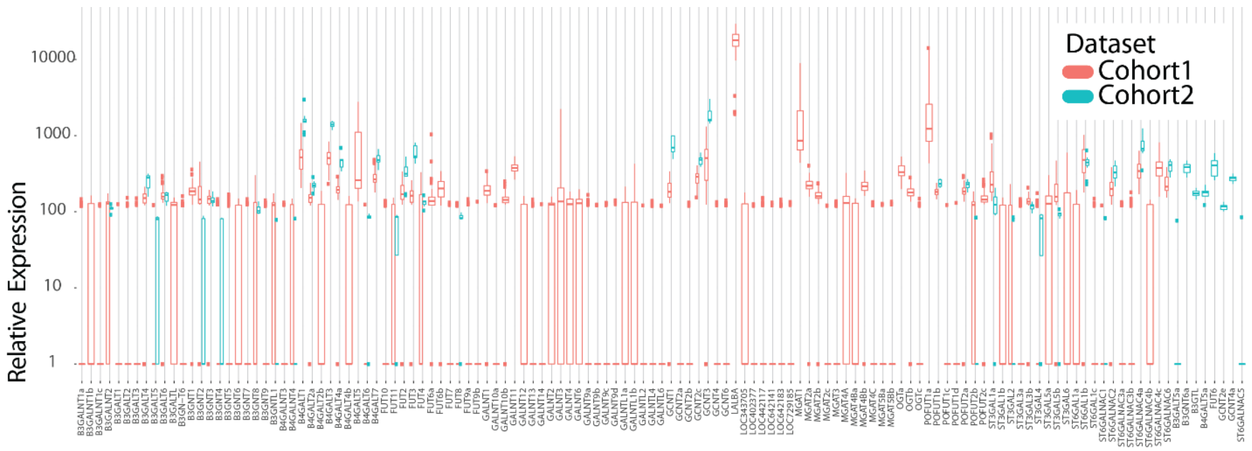

Supplementary Figure 1 - ***Distribution of Gene Expression Values of Each Gene Candidate.*** *Distribution of gene expression values from microarray experiments for each gene examined across all samples stratified between dataset1 (red) and dataset2 (blue). Cohort 1 includes 48 samples with 8 temporal observations per 6 subjects. Cohort 2 includes 10 samples with 2 temporal observations per 5 subjects. Data are presented as Tukey boxplots (median centerline, box spans the interquartile range (IQR), whiskers extend to +/- 1.5*IQR, outliers (>|1.5*IQR) are presented as points.*

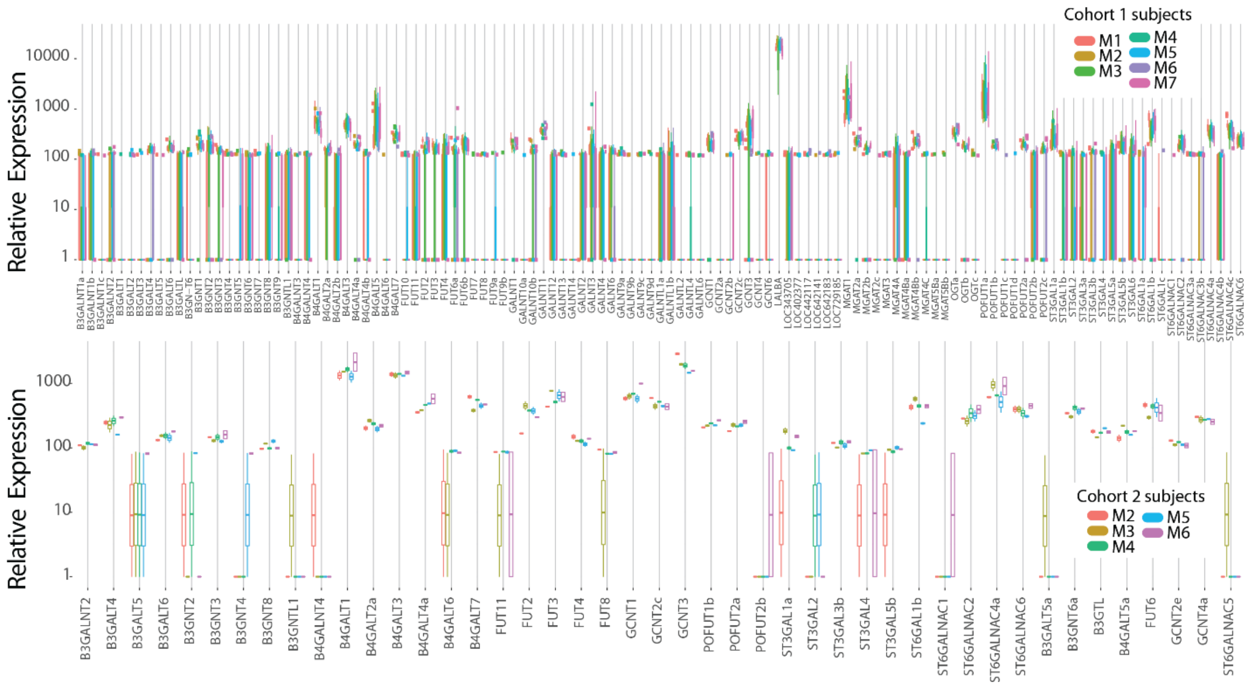

Supplementary Figure 2 - ***Distribution of Gene Expression Values of Each Gene Candidate Stratified by Subject.*** *Distribution of gene expression values from microarray experiments for each gene examined across all samples stratified between dataset (dataset1 (top) and dataset2 (bottom)), and subject (color). Cohort 1 (top) includes 48 samples with 8 temporal observations per 6 subjects. Cohort 2 (bottom) includes 10 samples with 2 temporal observations per 5 subjects. Data are presented as Tukey boxplots (median centerline, box spans the interquartile range (IQR), whiskers extend to +/- 1.5*IQR, outliers (>|1.5*IQR) are presented as points.*

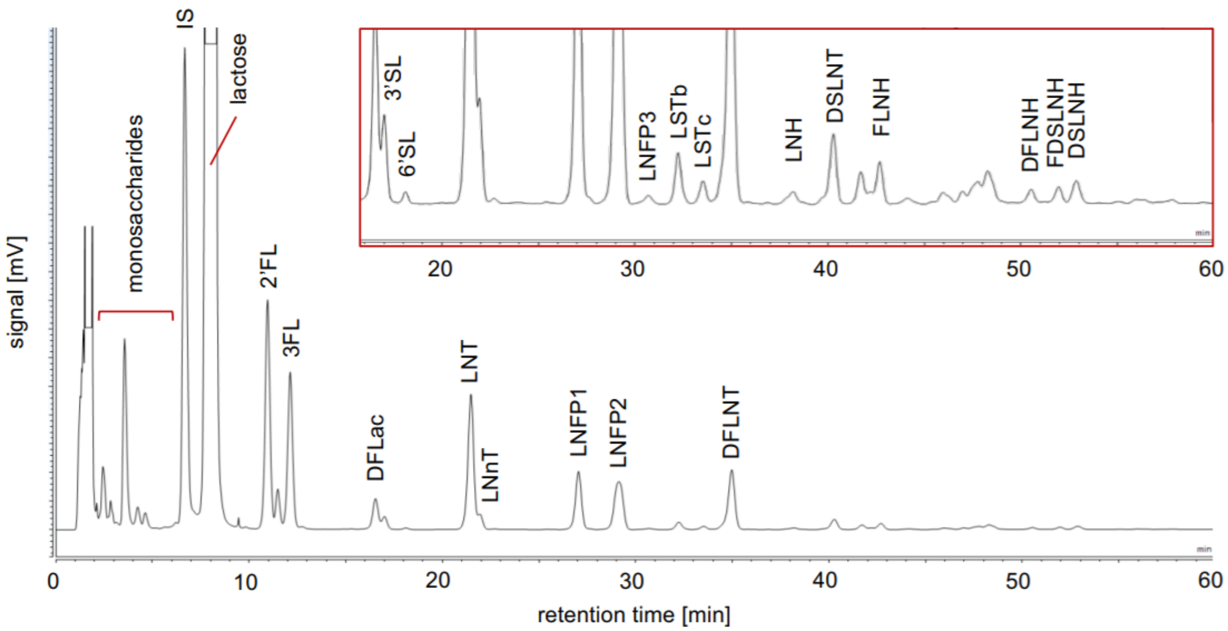

Supplementary Figure 3 - **HPLC-FL chromatogram of 2AB-labeled HMOs in human milk.** IS (internal standard) is added at the beginning of sample workup to allow for absolute quantification of HMOs relative to internal standard. Monosaccharides elute early while oligosaccharides elute later based on their molecular structure. Only HMOs that can be clearly distinguished are annotated and quantified. Chromatogram between 16 and 60 min is enlarged to better visualize minor peaks (box framed in red).

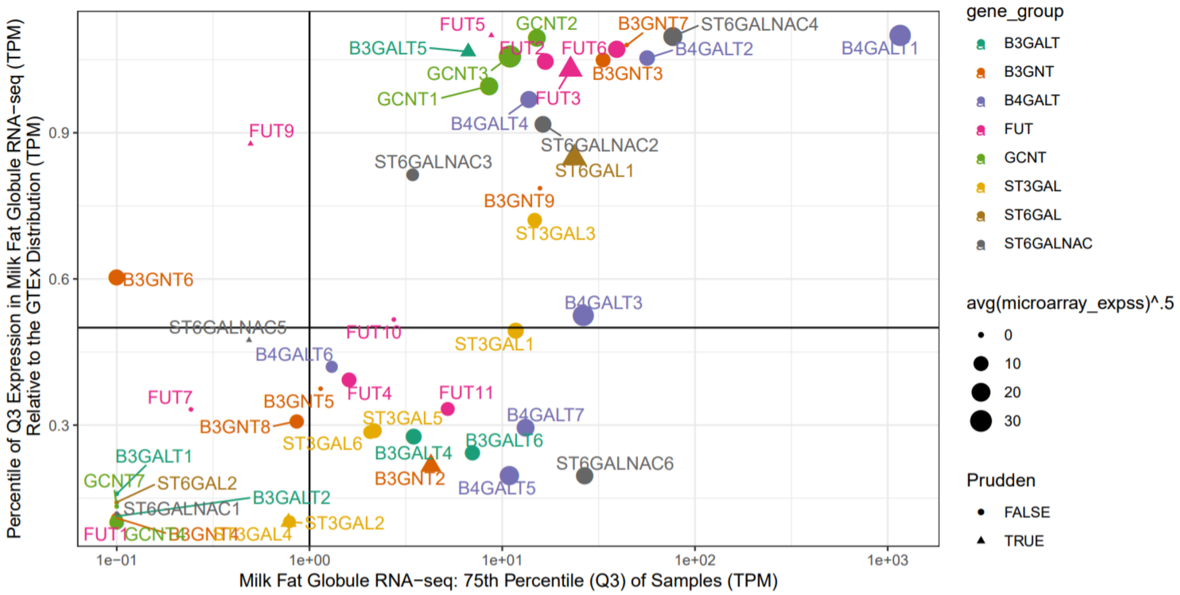

Supplementary Figure 4 - **Expression (75th percentile of TPMs, Q3) of glycosyltransferase genes in breast epithelium vs the percentile of Q3-TPM expression relative to GTEx expression**. The RNA-Seq (GSE45669[1]) was used to validate low expression in the microarrays (size). Genes used in Prudden et. al.[2] were highlighted with triangles.

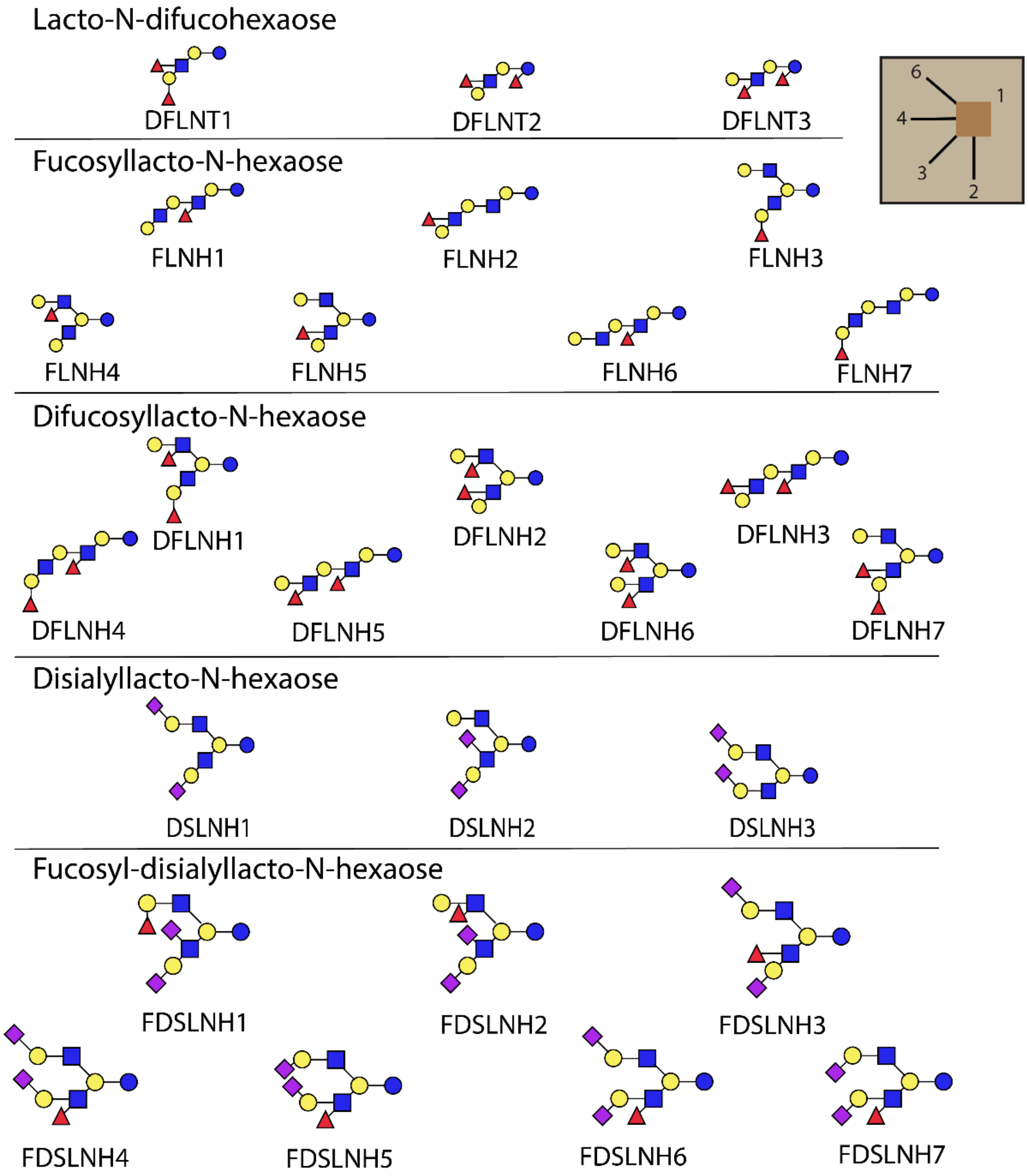

Supplementary Figure 5 - ***Candidate structures of uncharacterized human milk oligosaccharides.*** *5 HMOs quantified for this study are not structurally well-characterized. Each of these HMOs have been associated to candidates structures identified based on published work*[3]*. The structures of some HMOs are unclear. Due to the diversity of monosaccharides that could be attached through different linkages, a huge number of structurally distinct HMOs are possible. Of the many possible HMOs, more than 150 have been identified*[4]*. Several of the most abundant observed HMOs remain to have ambiguous structures. The natural heterogeneity (branching, isomerization and polarization) of HMO mixture present in milk makes their structural identification and quantitative detection a prohibitive challenge to many current studies*[3,5,6]*. This is due in part to the scarcity of standards. In this study we use High Performance Liquid Chromatography (HPLC) to measure the abundance of the 16 most abundant HMOs. Of the measured HMOs, 11 have fully determined molecular structures, while the remaining five have multiple candidate structures.*

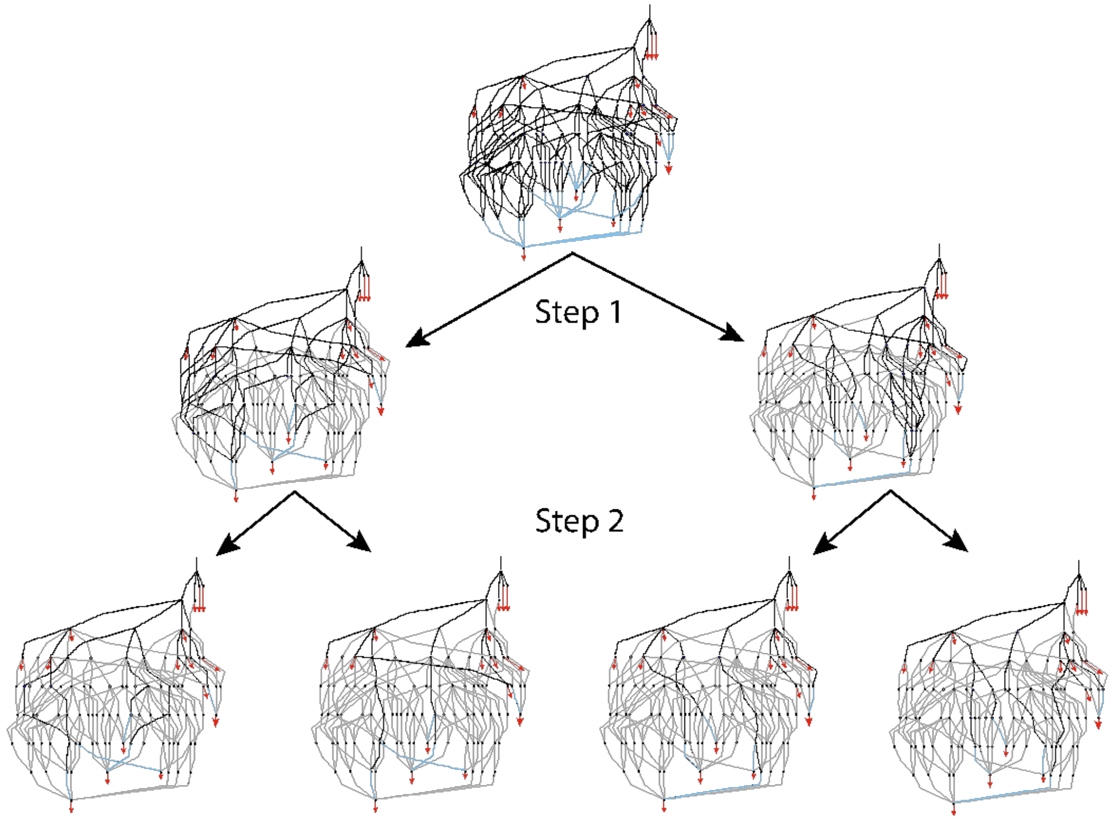

Supplementary Figure 6 - ***Subnetwork generation methodology.*** *Different combinations (i.e. only two combinations of the 5 uncharacterized HMOs are shown in the* ***Figure****) are fixed to tailor the solution space (step 1) and a MILP algorithm is further applied to these reduced networks to enumerate all alternate subnetworks (step 2, only two alternate subnetworks by combination are showed in this* ***Figure****) that can synthesize all HMOs of interest.*

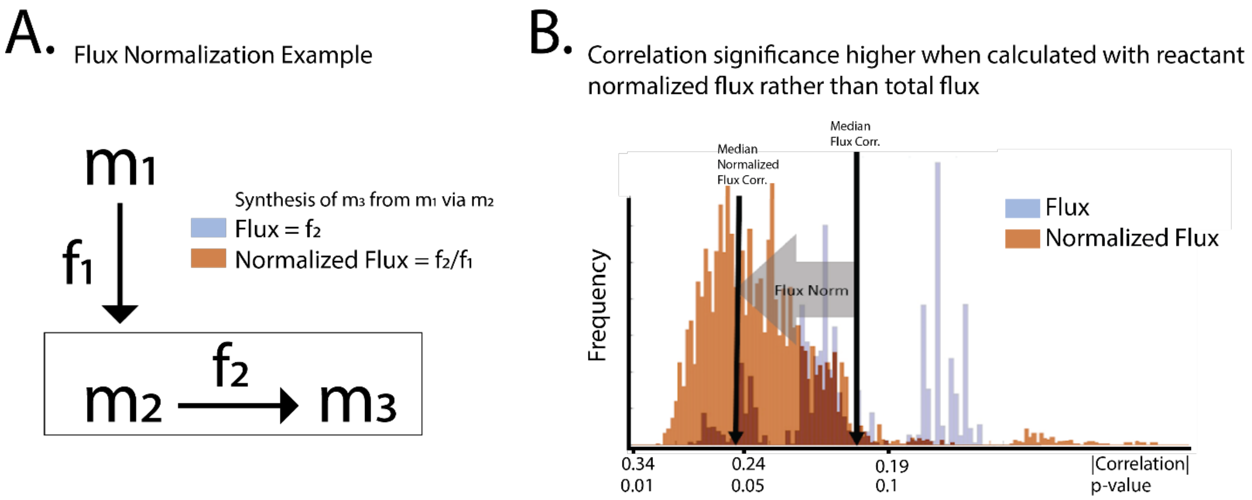

Supplementary Figure 7 - ***Comparison of total flux and normalized flux.*** *In this method we used prior-normalized flux to control for the availability of the precursor. Considering the enzyme abundance or presence can be informative but it is only one of the limiting factors in a synthesis reaction. Another essential factor to consider is the availability of the precursor.* ***(A)*** *shows the difference in calculating the total flux for reaction 2, conversion of m2 to m3. Total flux is contrasted by normalized flux f2/f1. This is to say that f2 can never exceed f1. If f1 is pulled to multiple reaction, this converts f2 to a measure of the proportion of m2 converted to m3.* ***(B)*** *shows the distribution of p-values for flux-expression Spearman correlations. Clearly, there is a shift towards significance when normalized flux is employed. In cohort 1, the maximum correlation of each linkage ranges from 0.0655 to 0.714 with a median 0.38. Because the p-value/correlation mapping is bijective, the x-axis indicates both p-values and correlation (cohort 1, n=48).*

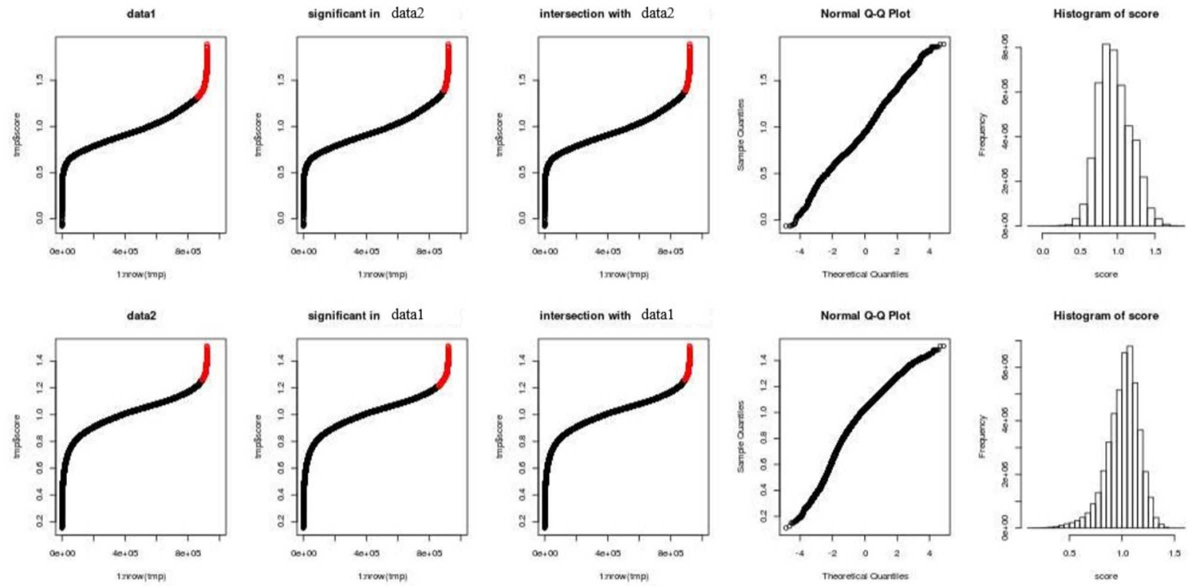

Supplementary Figure 8 - ***Selection of models presenting a high model score*** – *(lettered by row, left to right, up and down) A-C. Candidate Model Scores, $S_{\omega}(D_1)$, sorted by magnitude; sorted index vs. magnitude. F-H. Candidate Model Scores, $S_{\omega}(D_2)$, sorted by magnitude; sorted index vs. magnitude. A and F show the sorted scores for $S_{\omega}(D_1)$, and $S_{\omega}(2)$, respectively. Red points are top-performing candidate models. B and G show the top-performing models (red) from F and A respectively mapped onto the distributions from A and F to show cross-comparability. C and H show the commonly top-performing models (red) between dataset1 and dataset2 mapped onto score sigmoid 1 and 2 respectively to show the models in the intersection perform well. D and I show Q-Q plots of the score distributions to evaluate the normality of the distribution. E and J show distributions of the scores to further visualize normality of the distributions.*

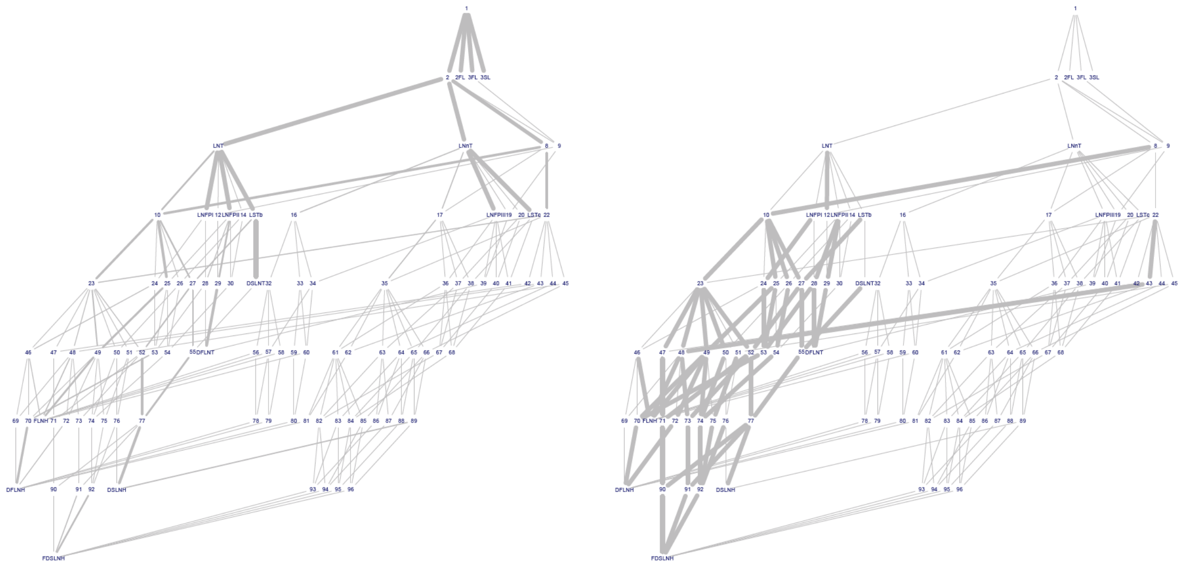

Supplementary Figure 9 ***- Reduced Network weighted by proportion and enrichment of each reaction in the high-performing models.*** *Edge thickness indicates proportion of top-performing models which include a reaction (left), or enrichment of reaction in the top-performing model set as compared to the background model set (right). Each node is numbered referring to an HMO* ***Supplementary Dataset 3****: HMOs.*

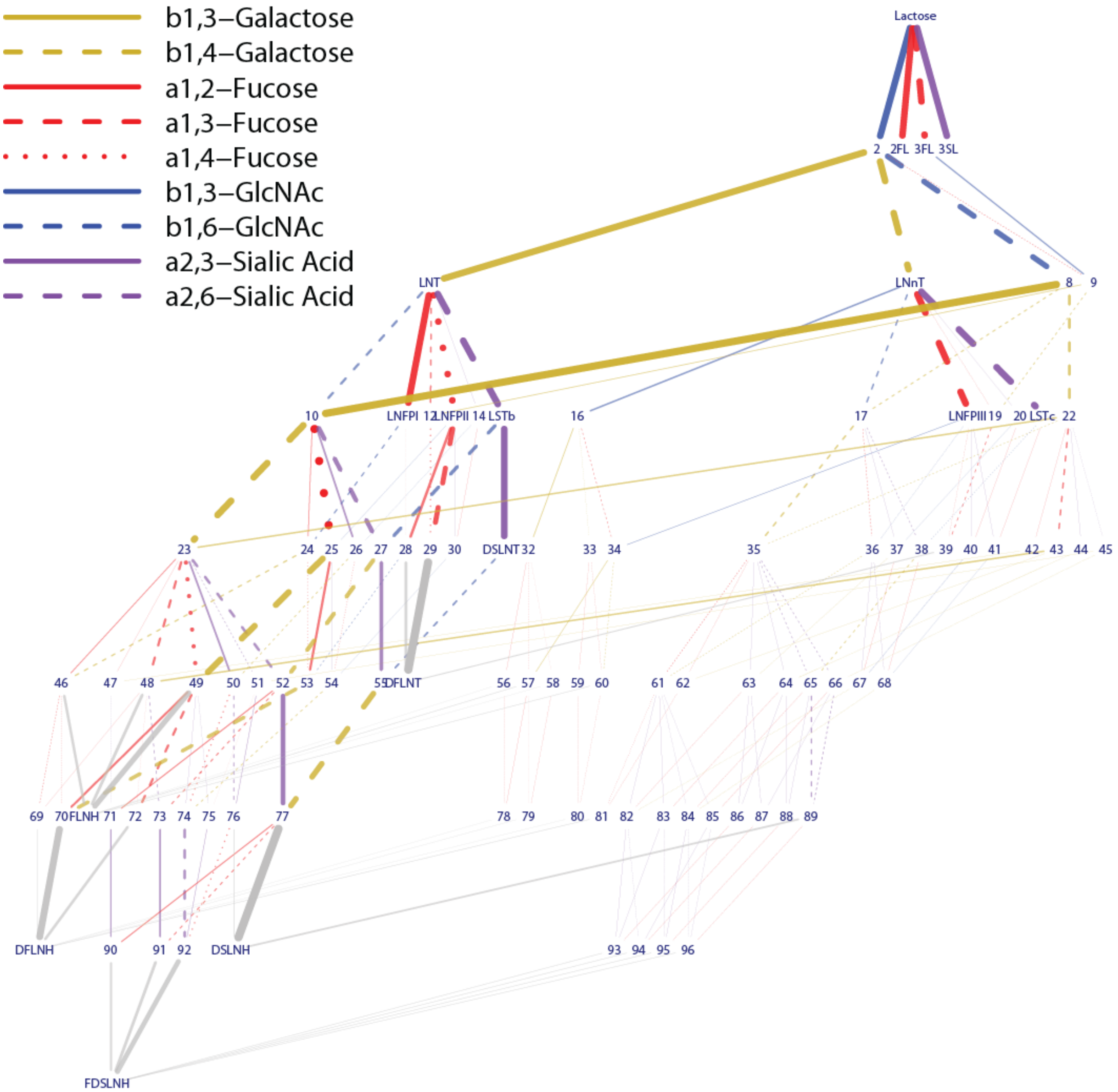

Supplementary Figure 10 - ***The reduced network produced by flux variability analysis.*** *The Reduced Network retains all reactions from the Complete Network,* ***Figure*** *3B, necessary to perfectly predict the HMO profiles collected by High Throughput Liquid Chromatography (HPLC). Numerical nodes are unmeasured intermediary HMOs specified in* ***Supplementary Dataset 3****.*

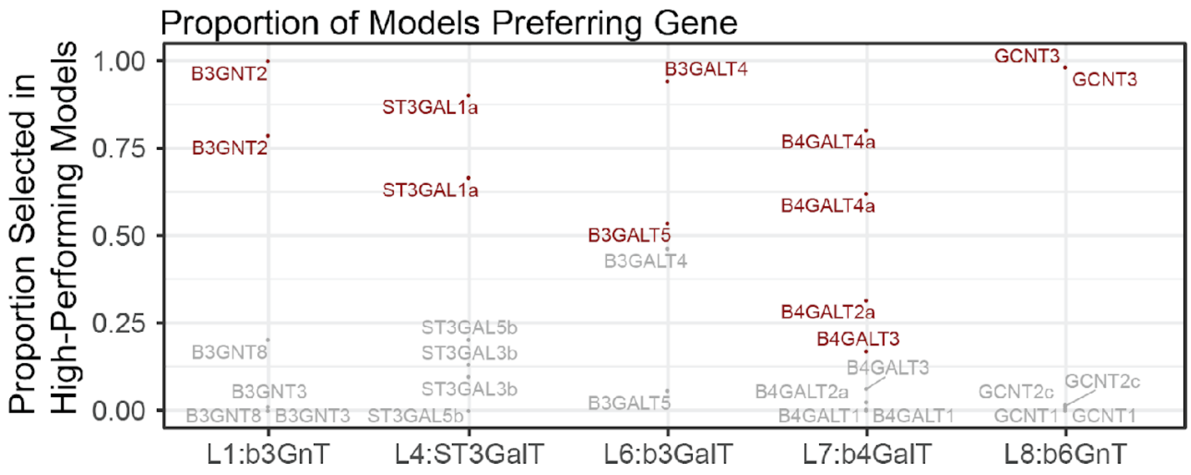

Supplementary Figure 11 **– *Gene expression correlation with model flux predicts enzymes involved in HMO biosynthesis.*** *The proportion of commonly high-performing models where each isoform was most correlated to the model predicted flux. Significance (p<0.001, red) is calculated by hypergeometric enrichment of representation in high-performing models compared to all models. Each gene appears twice since data are analyzed separately for the two cohorts. For exact P values of hypergeometric enrichment, see* ***Source Data****.*

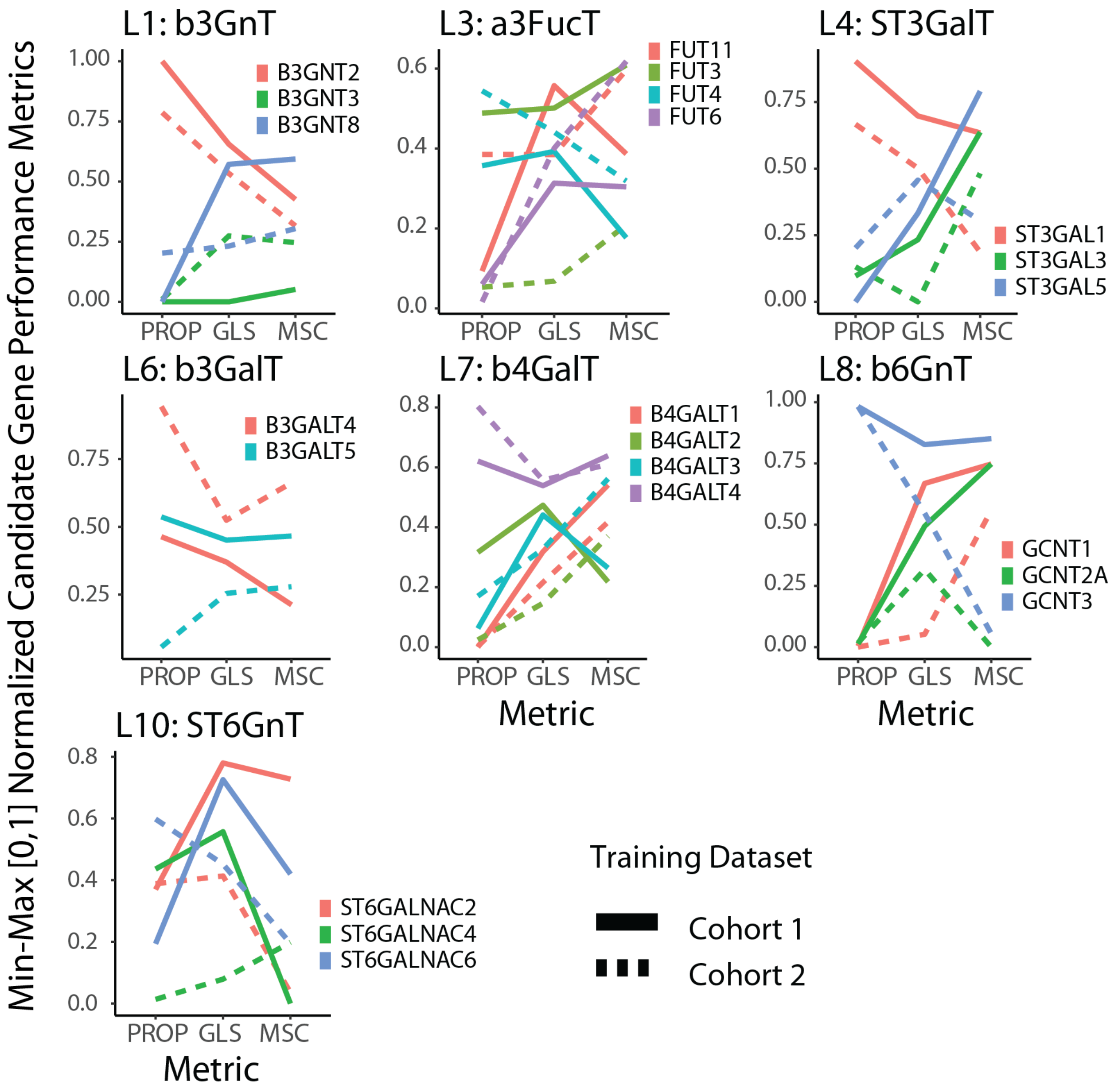

Supplementary Figure 12 ***- Proportion (Prop), Gene Linkage Score (GLS) and Model Score Contribution (MSC) for each candidate gene within each gene linkage.*** *Each panel corresponds to one major linkage type and shows the three performance metrics (x-axis) for all considered isoforms (line color) evaluated on data from either cohort 1 or cohort 2 (line type). The y-axis describes the actual value of each metric min-max normalized between zero.. Please refer to Section 2.2 and 7.7 in main text for a detailed description on how these gene performance metrics were used for gene selection.*

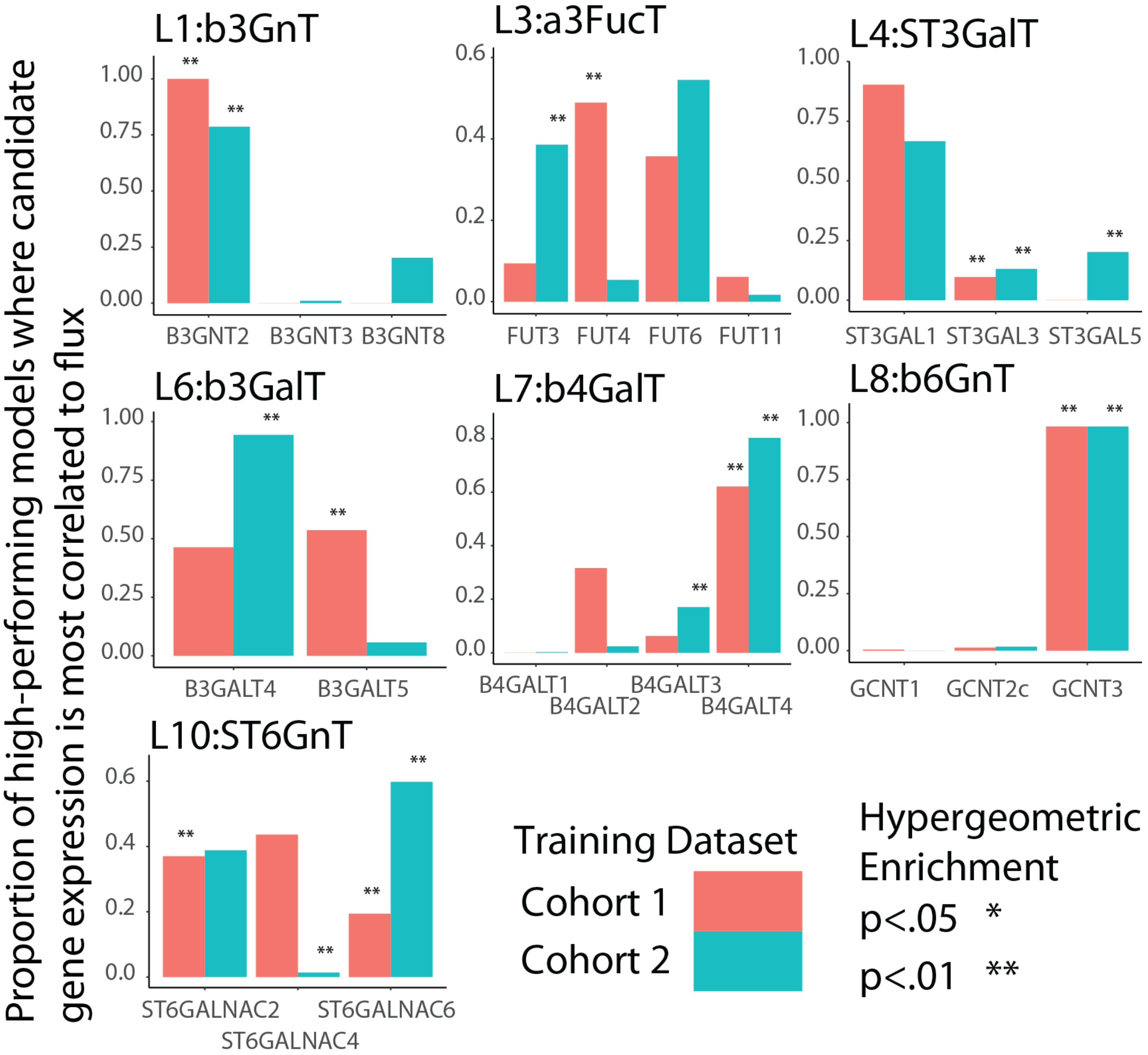

Supplementary Figure 13 - ***Proportion of Models Where Expression of a Given Gene is Most Consistent with Predicted Flux.*** *Gene Expression was measured in dataset 1 (red) or dataset 2 (blue) then compared to normalized flux predicted for models commonly high-performing with cohort1 and cohort2 data as described in Methods section Ambiguous Gene Selection and Supplemental Methods section 1.1.5). For exact P values of hypergeometric enrichment, see* ***Source Data****.*

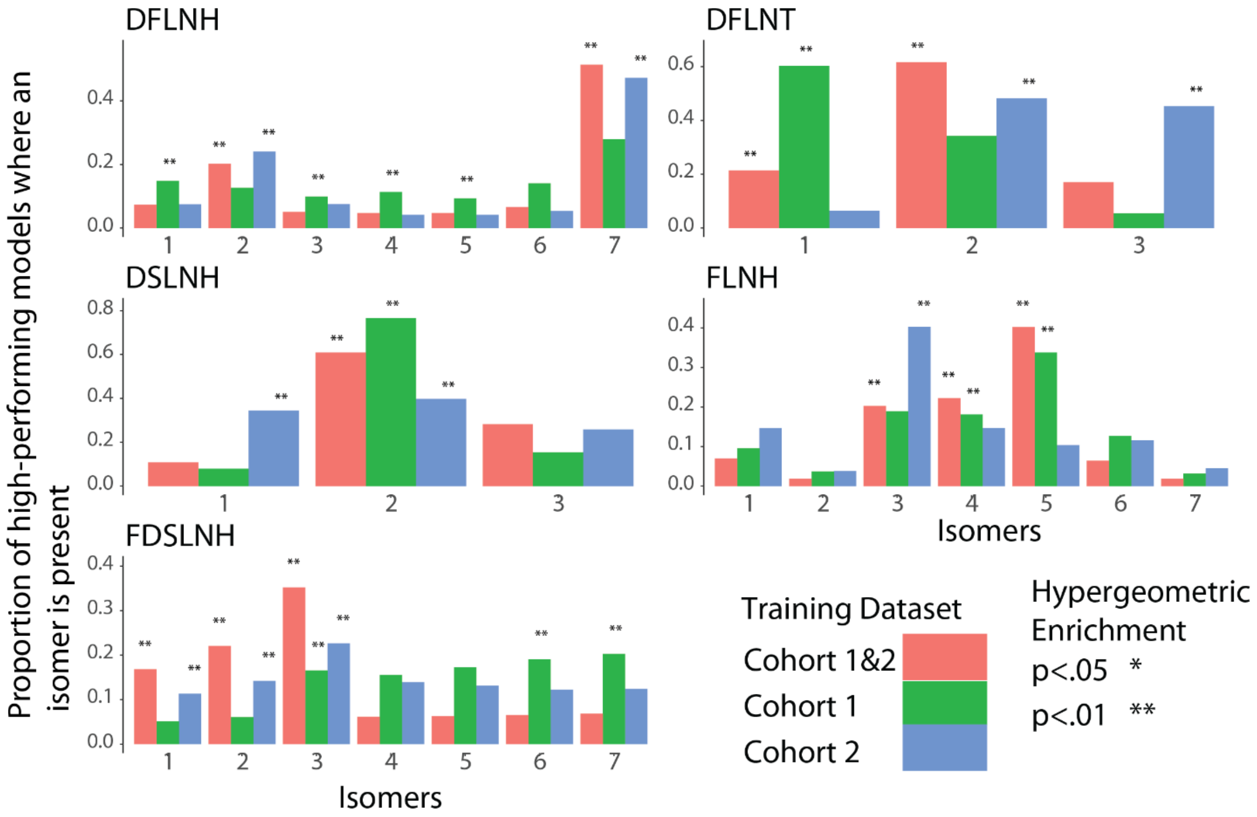

Supplementary Figure 14 - ***Proportion of Each Structure Isoform Appearing in Top-performing Models.*** *Barplots describing the proportion of models in top-performing model sets containing each isoform of an ambiguous HMO structure. Top-performing model sets include those top-performing when parameterized on cohort 1 data (red), models that performed well with data from cohort 2 data (green), and models that performed well in both cohort 1 and cohort 2 (blue). Specific structures are illustrated in* ***Supplementary Figure*** *5. For exact P values of hypergeometric enrichment, see* ***Source Data****.*

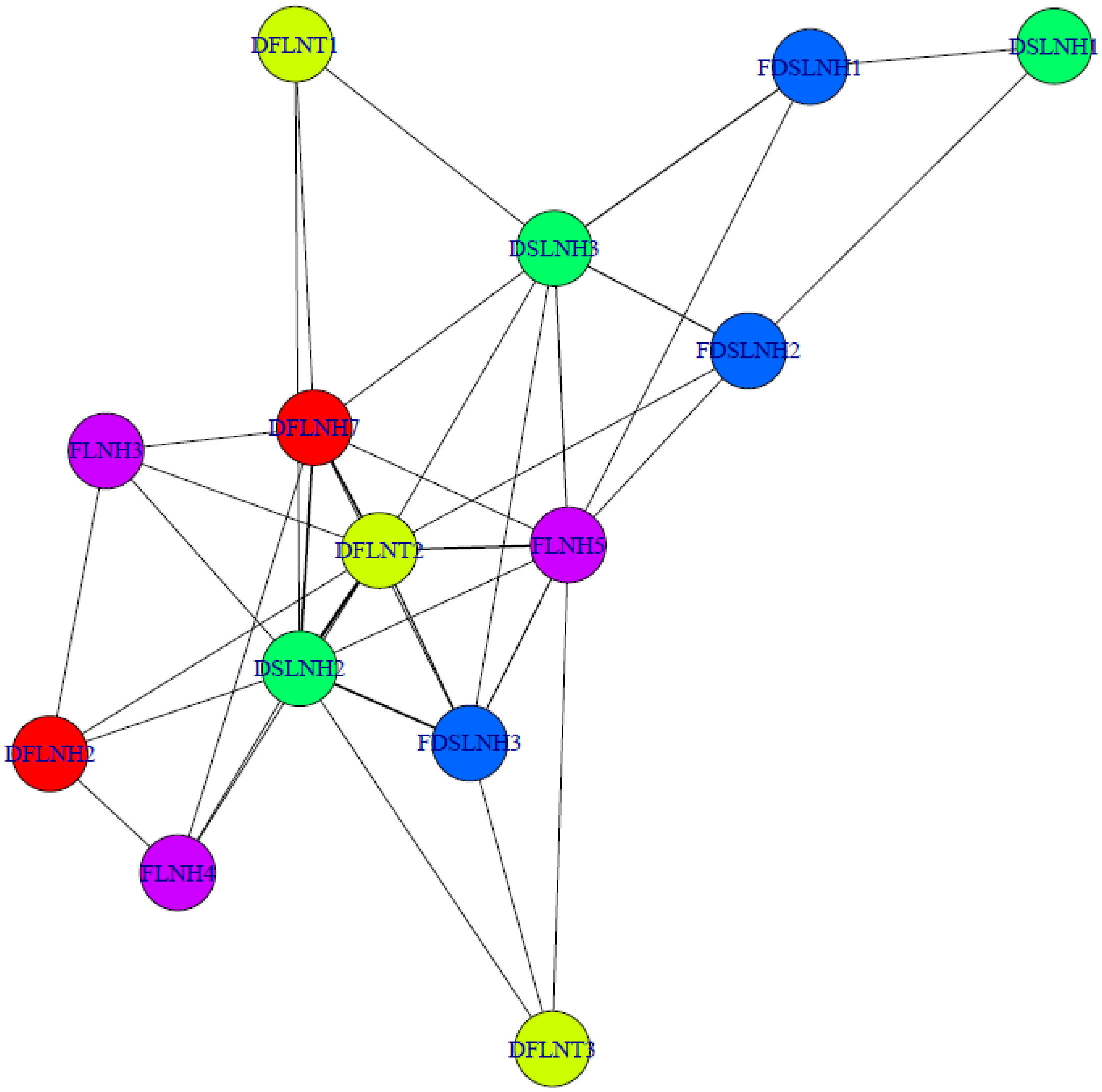

Supplementary Figure 15 ***- Jaccard Structure Co-occurrence.*** *Network of the Jaccard index measuring co-occurrence of each pair of ambiguous HMOs structure within the set of best performing models. For exact numbers, see* ***Supplementary Dataset 3****.*

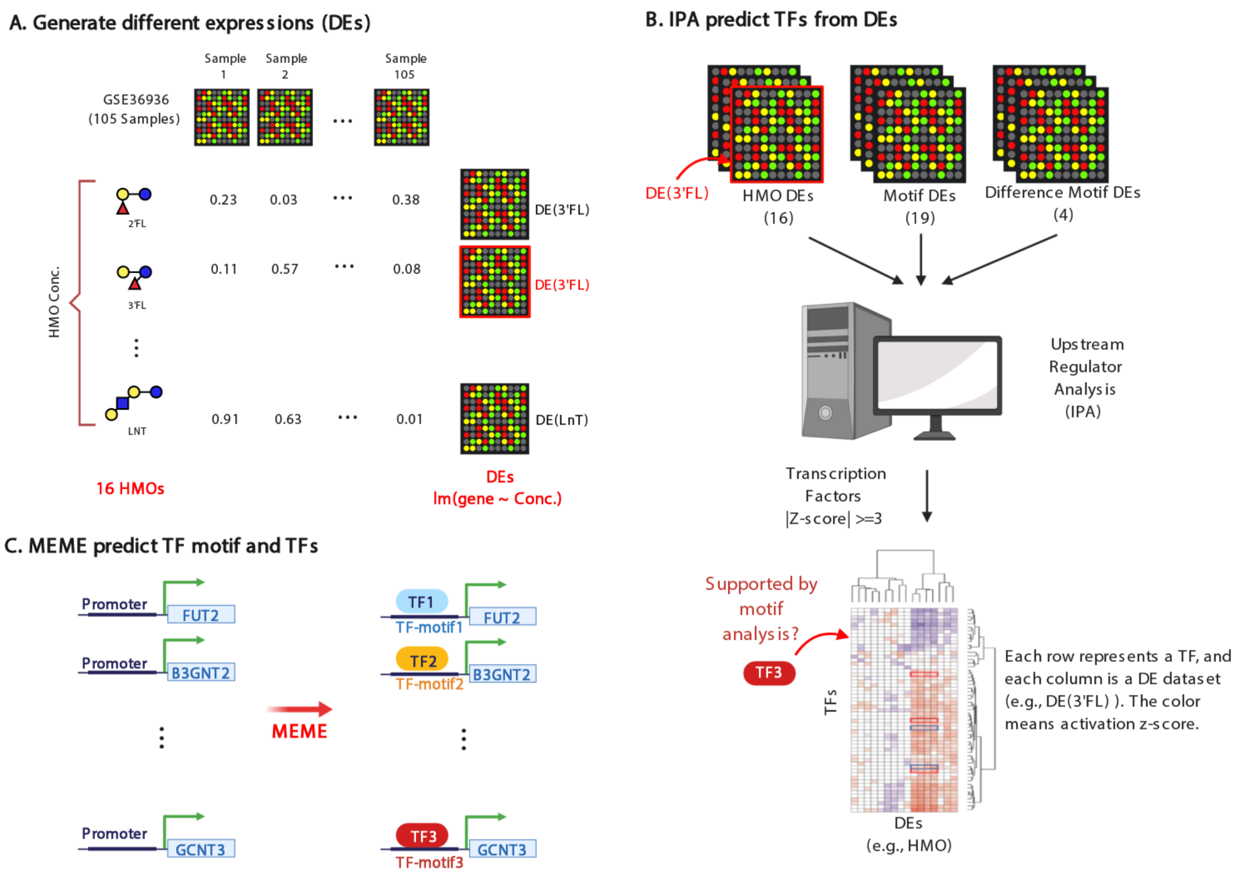

Supplementary Figure 16 - ***Procedures of the Transcriptional Factor Analysis. (A)*** *We first conducted 39 differential expression (DE) analyses to identify gene expression associated with variance in glycan and motif abundance (e.g. limma(expression ~ [3FL])). The 39 abundance measures include: 16 HMO concentrations, 19 glycan motif abundances, and 4 glycan motif abundance ratios.* ***(B)*** *We used IPA to predict transcriptional factors (TFs) that could explain differential expression associated with changes in HMO and motif abundance.* ***(C)*** *To corroborate the identified TFs, we used MEME to perform TFs binding site analysis for the glycosyltransferases (GTs) based on their promoter sequences.*

(A) TFs of HMO, Motif and Differential Motif

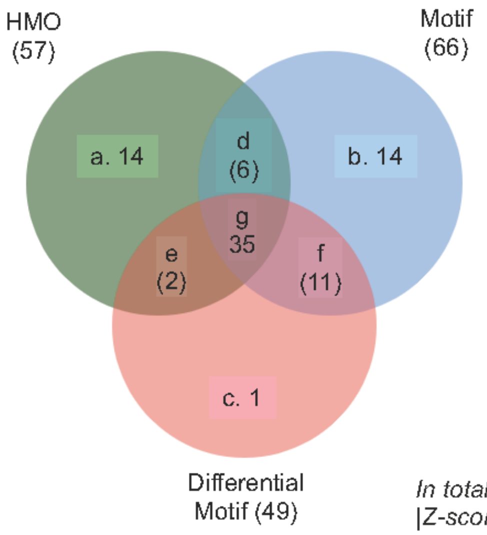

In total, there are 83 TFs.
|Z-score| >= 3 (IPA)

(B) Breakdown of the total 83 identified TFs

(a) HMO unique (14)

ETV4, IKZF2, IRF2, KAT5, MED1
MITF, MLXIPL, NANOG, NCOA3, POU2F2
REL, SMARCB1, SREBF1, ***STAT5A***.

(d) HMO and Motif shared (6)

E2F1, ERG, FOS, JUNB, ***NFKBIA***,
SP1

(b) Motif unique (14)

ATF4, E2F3, ELK1, HLX, IKZF3
KLF3, LHX1, MTPN, PLAG1, RB1
TAL1, TBX2, TBX21, XBP1

(e) HMO and Diff. Motif shared (2)

FOXM1, FOXO1

(c) Differential motif unique (1)

PHF12

(f) Differential motif and Motif shared (11)

CDKN2A, FOXL2, IKZF1, JUN, MYC
MYCN, NFAT5, NKX2-3, SMAD3, ***STAT3***
TP53

(g) HMO, Motif, and Differential Motif shared (35)

BCL3, BCL6, ***CEBPA***, ***CEBPB***, ***CREB1***, ECSIT, EGR1, ETS1, ***GFI1***, GPS2
HIF1A, HMGB1, IFI16, ***IRF1***, ***IRF3***, ***IRF5***, ***IRF7***, ***NFKB1***, ***NFKB2***, NUPR1
PML, PRDM16, ***RELA***, ***RELB***, SIRT1, SMARCA4, SP110, SPI1, SREBF2, ***STAT1***
STAT2, ***STAT4***, TFEB, ***TRIM24***, ZFP36

Many of the HMO, motif, and differential motif shared TFs (~50%; highlighted in the blue colors) are involved in immune-related pathways, e.g., type-I/II interferon signaling pathways.

Supplementary Figure 17 - ***Transcriptional factors (TFs) identified by IPA using the gene expression data.*** *(A) Based on the Z-score predicted by IPA, we identified a total of 83 significant TFs with |Z value|>=3 in this study. (B) We show the breakdown of these 83 TFs into different categories.*

A. TF motif – I

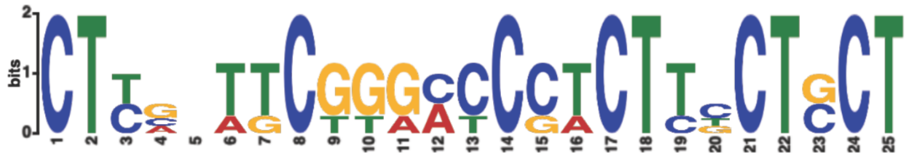

GlycosylT.	Strand	Start	P-value	Sites
GALNTL6	+	76	2.56e-13	tcagctctgtCTTGCTTCGGGATCCTCTTCCTCCT
ST6GALNAC6	+	46	1.14e-11	caaggcctgaCTTCTTTCTGGCCCCACTTTCTGCTgtagtggtct
→ ST3GAL1	+	14	2.76e-11	ctgccatcctCTCGGTGCGTGCCCGTCTTCCTGCTgaaacggacc
OGT	+	35	4.29e-11	tgaagacgggCTCAAATCGGAACCCTCTCGCTCCTcgttttgagc

B. TF motif – II

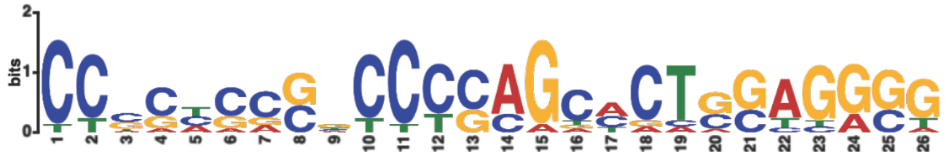

GlycosylT.	Strand	Start	P-value	Sites
→ B3GNT2	+	72	1.39e-12	cggggccattCCTCACCCGCCCCAGCACTGGAGGGGgcg
→ B4GALNT4	+	12	7.67e-11	acagctcggcCCCCTGCGGCCCCAGCCCTGCAGGCTgagatcccgc
POFUT2	+	22	2.71e-9	tgcgcggctcCCGCCCCGACCCCAGCTCCAGAGGGGcgagagcacc
POFUT1	-	45	3.01e-9	AGCCACCGGTCCCCTCCCTTCCGAGACCTGGAGACGCAGTCCTGAG
GALNT9	+	54	4.12e-8	gggactcagcTTCCCCAGTCCTCAGCCCTGGACGGGggagagggag
MGAT3	-	69	5.61e-8	ACATGCCCGAAGCCACCTGCGCACTCCTGGGGTAACTGGGGG
GALNT2	+	66	7.54e-8	agcaccgaggCCACTACCGCCCCCGTAATCGAGAGGccccgtgtg
→ FUT11	+	39	1.00e-7	tcctttccagCTGGAGAGGCCCCAACACTGGATGGTtctgtaggga
→ ST6GALNAC2	+	32	1.08e-7	ctccaccctgCCCGCCCCCTCCCAGCTGTCCTGGCGtccgcagact
B4GALT6	-	18	1.62e-7	GTGAGGCGCCCCCGCCGGGCCCGCGGACACCAGGCGAAGAGCAACC
B3GALT6	-	60	3.03e-7	CATCGTCCGTCCGCTCGGCCTTGAGCCCTGGCGAGACTCCGCCGCC

C. TF motif – III

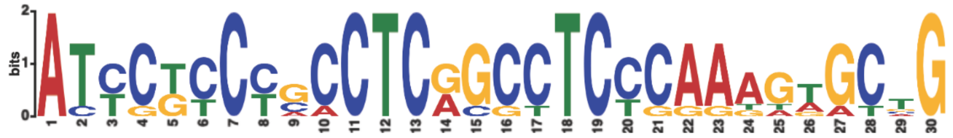

GlycosylT.	Strand	Start	P-value	Sites
B4GALT7	+	14	1.77e-17	tgacctcgtgATCCGCCCGCCTCGGCCTCCCAAAGTGCTGggattacagg
MGAT1	+	2	1.23e-16	gATCCGTCTGCCTCGGCCTCCCAAAGTGCTGggattacagg
→ FUT2	-	11	3.63e-16	AGTTCAAGGGATTCTCCTGCCTCAGCTTCCCAAAGTGCTGGGATTACAGA
FUT6	+	45	1.75e-14	ggttcacaccATTCTCCTGCCTCAGCCTCCCAAGTAGCTGggactacagg
B3GNT3	+	27	1.55e-12	ggttccagcgATCCGCCCCCCTCGGCCTCTGGAAGTACAGgagtgagcca
FUT10	+	58	6.86e-12	gactcaagagATCCTCCCACCTCAGCCTCCCAGTAGGTGGgactacaggc
ST3GAL2	-	27	2.69e-11	GCCGGAGGGCACTGTTCCCACTCGCGCTCTCAAAGAGCCGCGACCTGAGG

Supplementary Figure 18 – ***TF motifs identified by MEME using the promoter sequence of GTs.*** *There are three TF motifs that their known TFs overlapped with the IPA predict TFs: (A) TF motif-I. (B) TF motif-II, and (C) TF motif-III. The red arrows highlighted GTs are those major GTs involved in the HMO biosynthesis selected in this study (**Figure 5C** in the main text). The p-value represents the significance of the MEME identified TF motif associated with the GT by the aligned potential TF binding site on the promoter sequence. P values are determined by log likelihood ratio (as described by Bailey & Elkan[7]).*

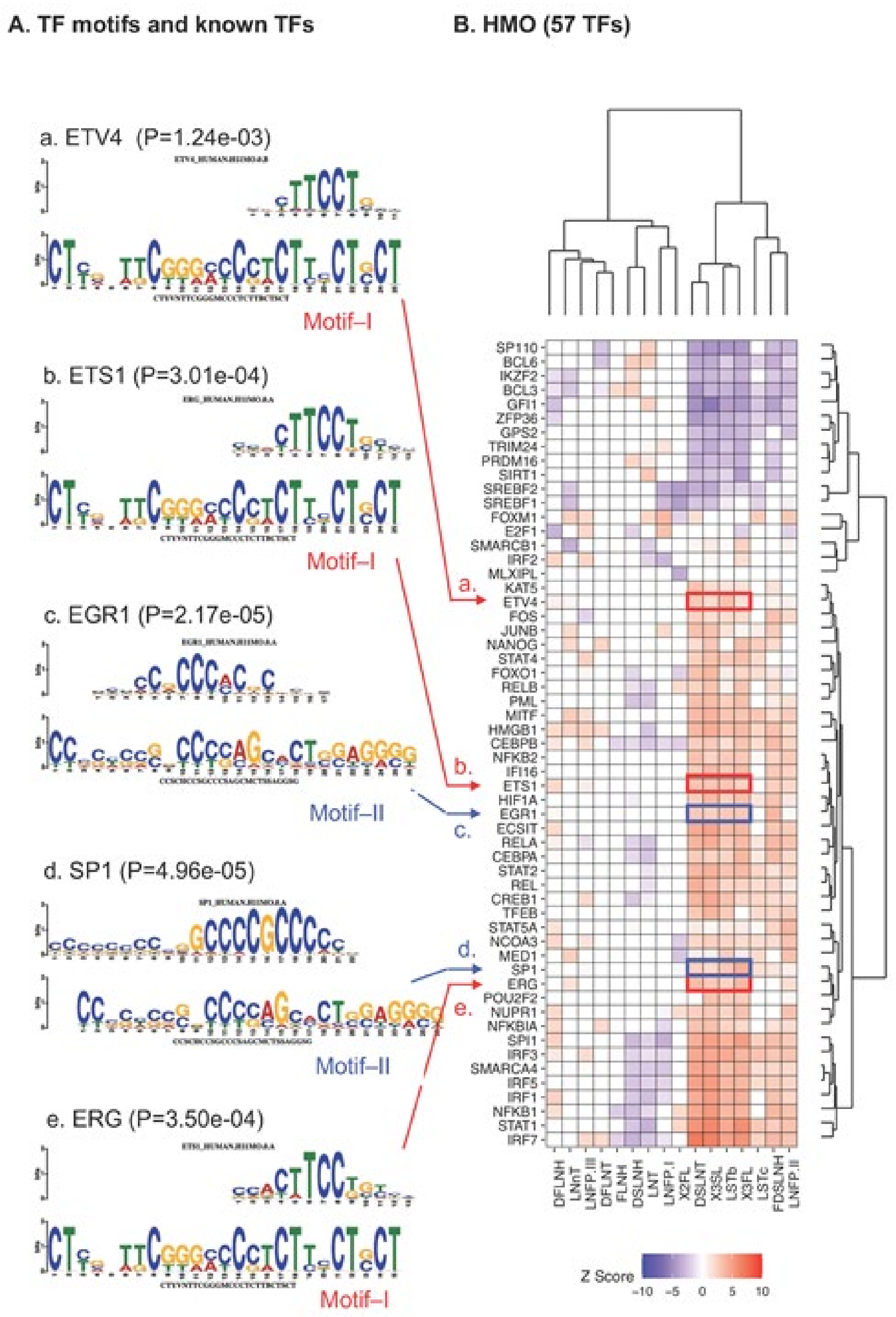

Supplementary Figure 19 – ***MEME promoter-enriched TF motifs and IPA predicted TFs using differential expression analyses with respect to 16 HMOs.*** *(A) MEME identified TF motifs and 5 known TFs (ETV4, ETS1, EGR1, SP1, and ERG) associated with them (see* ***Supplementary Table 4****). MEME-discovered TFs were cross-referenced with known TF binding sites using TOMTOM. Logos for the matched known and discovered motifs are shown in the top and bottom of each subpanel (A.a-e). P value describe the significance of a MEME logo match to known motifs calculated by TOMTOM (as described in Gupta et al.[8]). (B) Biclustering of activation z-score computed by IPA indicating the likelihood that a TF activates (z>0) or inhibits (z<0) an HMO concentration signature (gene expression associated with changes in HMO concentration).*

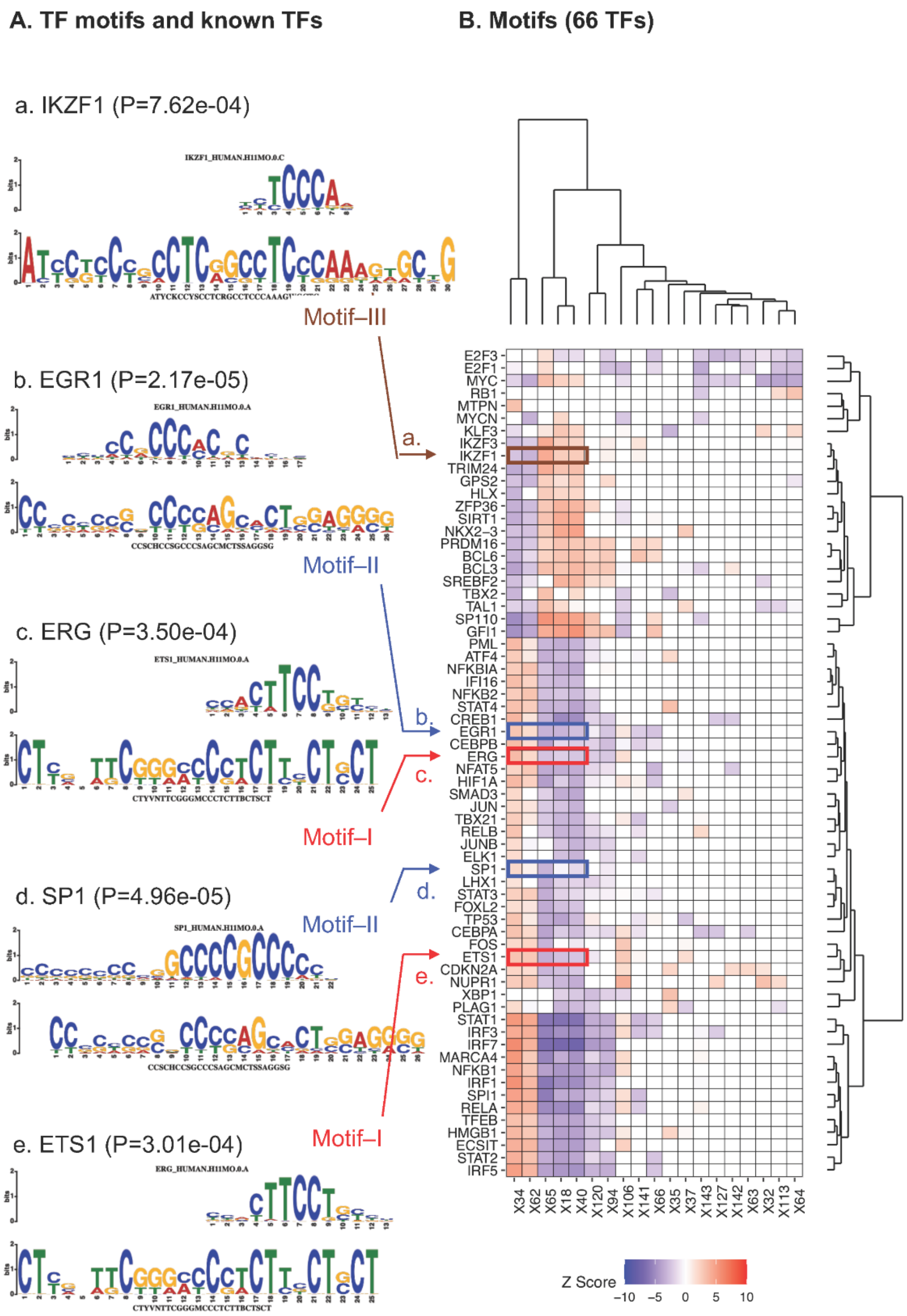

Supplementary Figure 20 – ***Clustering results and MEME identified TFs of the IPA predicted TFs using the gene expression data (19 Motifs).*** *(A) MEME identified TF motifs and 5 know TFs (IKZF1, EGR1, ERG, SP1, and ETS1) associated with them (see* ***Supplementary Dataset 2****). The known TF binding site motifs discovered by TOMTOM is given on the first line of each box, followed by the motif discovered by MEME, all shown as aligned logos. P value describe the significance of a MEME logo match to known motifs calculated by TOMTOM (as described in Gupta et al.[8]). (B) Clustering results of the IPA identified TFs using the 19 motif DE data. The color in the heatmap denotes the IPA predicted activation (Z-score) of each TF in the indicated glycan motif. X[number] glycan substructures defined in* ***Supplementary Figure*** *21.*

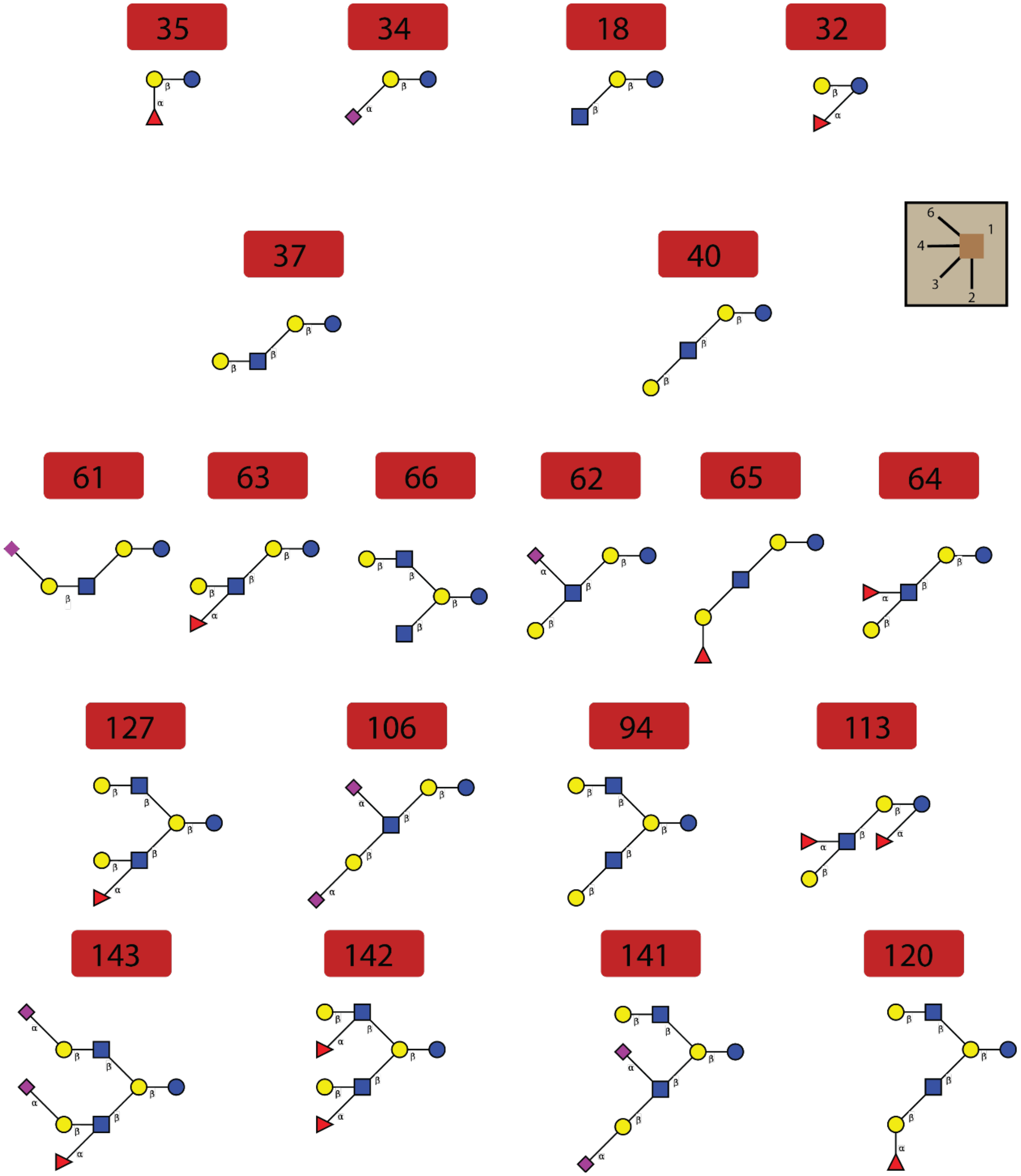

Supplementary Figure 21 **- *HMO substructure network with dependent substructure removed.*** *All the glyco-motifs examined in the IPA transcription factor analysis. Reproduced with permission*[9]

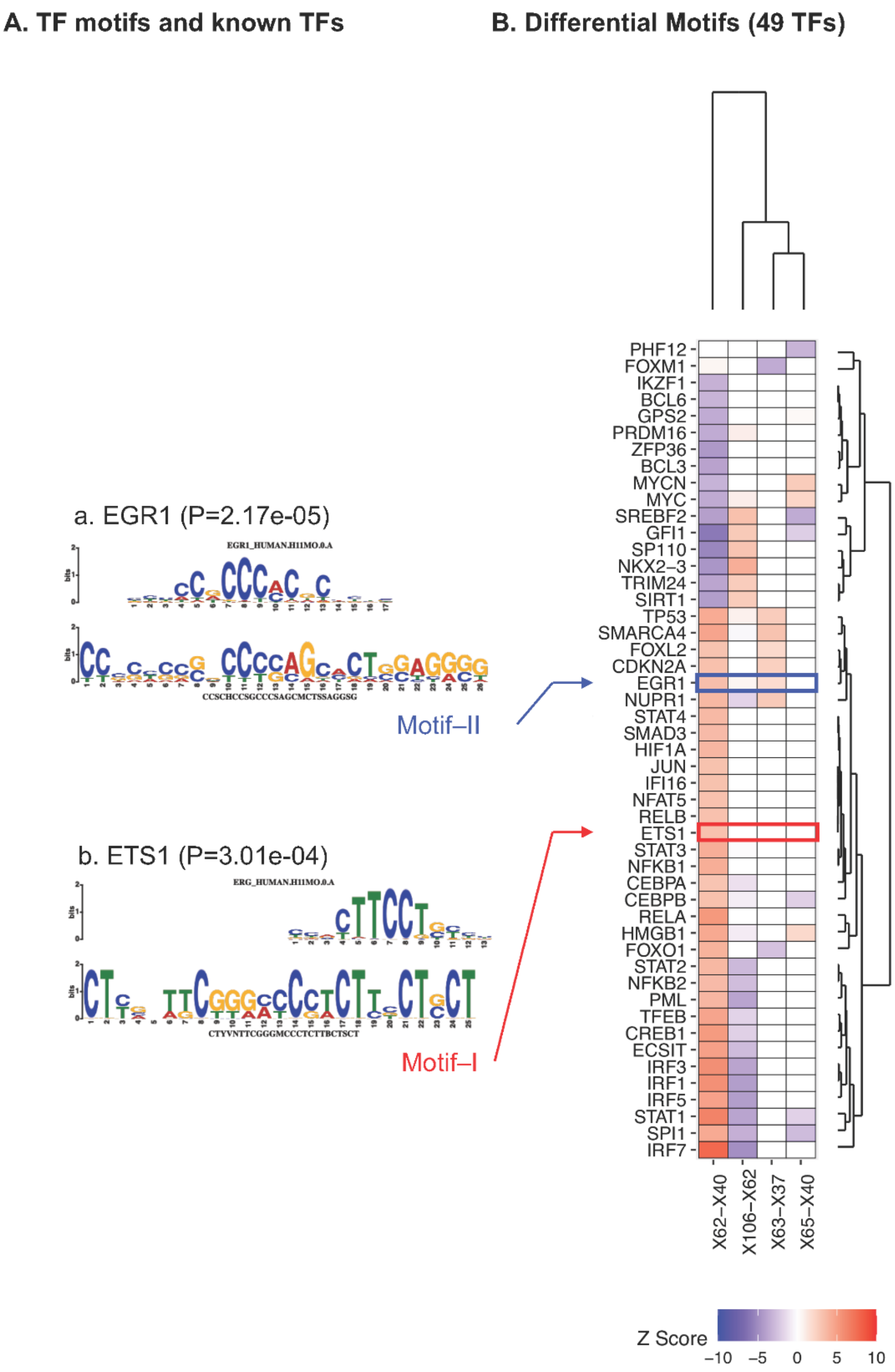

Supplementary Figure 22 – ***Clustering results and MEME identified TFs of the IPA predicted TFs using the gene expression data (4 differential motifs).*** *(A) MEME identified TF motifs and two know TFs (EGR1 and ETS1) associated with them (see* ***Supplementary Dataset 2****). The known TF binding site motifs discovered by TOMTOM is given on the first line of each box, followed by the motif discovered by MEME, all shown as aligned logos. P value describe the significance of a MEME logo match to known motifs calculated by TOMTOM (as described in Gupta et al.[8]) (B) Clustering results of the IPA identified TFs using the differential motifs DE data. The color in the heatmap denotes the IPA predicted activation (Z-*

score) of each TF in the indicated differential motif. X[number] glycan substructures defined in ***Supplementary Figure 21****.*

3 SUPPLEMENTARY DATASETS AND TABLES

Supplementary Dataset 1 **– *HPLC and microarray data used for modeling.*** *See methods for complete description of each dataset and cohort*

See associated file: Supplementary Dataset 1

Supplementary Dataset 2 **– *Linkages and genes considered throughout this analysis.*** *List of glycosyltransferase genes associated with each reaction and associated expression in datasets within and beyond this work. Here we list the 56 candidate glycosyltransferase genes considered. Columns* ***A-E*** *indicate the corresponding reaction, gene name and various identifiers. Column* ***G****, "Prudden," indicates if the gene was used Prudden et. al. 2017. Columns* ***H-J*** *show expression on cohort 1 and 2 microarrays (relative abundance) and an independent RNA-seq in the same tissue (TPM). Column* ***M-O*** *specify the distribution of TPMs in GTEx (min, Q1, Q2, Q3, Q4, max), the percentile where Q3 from the RNA-seq falls in the GTEx distribution, and the log2 Fold Change of RNA-seq Q3 vs GTEx Q3. Column* ***P*** *specifies any low expression events. Columns* ***Q-S*** *compare microarray and RNA-seq expression and usage in Prudden et. al. 2017. Column* ***T*** *indicates if previous literature found a gene relevant ("yes"), partially relevant ("similar") or irrelevant ("no") to the reaction in question; elaborated in* ***Supplementary Dataset 2****. Columns* ***U-V*** *delimited and justify which genes will be included for further analysis. The remailing columns provide some relevant detail on substrate specificity for each gene.*

See associated file: Supplementary Dataset 2

Supplementary Dataset 3 **- *The FVA reduced HMO biosynthetic network including HMO and reaction calculation referenced throughout the manuscript***

See associated file: Supplementary Dataset 3

Supplementary Table 1 - ***Reduction in model complexity across various examined models.***

model Size	Complete	Reduced	Candidate
HMOs	2,961	101	27-38
Reactions	9,918	221	43-54

Supplementary Table 2 ***- Number of total subnetworks High-performing candidate models from given dataset 1 or 2 with all sample or just secretor samples from the background of 44,984,988 subnetwork models.***

Data	Dataset 1	Dataset 2	Intersection
All Data	2,658,052	2,322,262	241,589

Supplementary Table 3 – ***Results of the NTP-Glo™ Glycosyltransferase Assay (Promega) to test GT candidates on relevant HMO acceptors.*** *++ indicates a strong effect, + indicates a weak effect, - indicates an insignificant effect, a blank cell indicates a negligible effect and a grey cell indicates an unmeasured reaction.*

Linkage	Reaction	Enzyme	Lactose	LNT	LNnT	Gal b-1,3-GalNAc
L1 b3GnT	b-1,3 N-acetylglucosamine	B3GNT2	++		++	
L4 ST3Gal T	(b-Gal) a-2,3 sialytransferase	ST3GAL1		+		++
		ST3GAL2		-		+
		ST3GAL3		++	++	+
		ST3GAL4		-	+	-
		ST3GAL5				
		ST3GAL6		+	++	-
L10 ST6GnT	(b-1,3-GlcNac) a-2,6 sialytransferase	ST6GALNAC 2				
		ST6GALNAC 5		-		

Supplementary Table 4 – ***MEME discovered 3 novel TF motifs and their associated known TF motifs that were supported by IPA predicted TFs***

MEME TF binding site motif	Known TF	Known TF binding site consensus	Overlap	MEME Mixture Model Likelihood p-value [*1]
**TF motif – I** 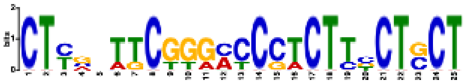	ETV5	CTCACTTCCTGCTC	13	6.74E-06
	ELF5	CCACTTCCTCCTTCC	12	1.78E-04
	ETS2	TCCTCTTCCTTCC	13	2.58E-04
	**ERG[*2]**	**CCACTTCCTGCCC**	**12**	**3.01E-04**
	**ETS1[*2]**	**CCACTTCCTGTCT**	**12**	**3.49E-04**
	ELF2	CCACTTCCGGGTT	12	6.75E-04
	EHF	GCCACTTCCTGGTTC	13	1.03E-03
	**ETV4[*2]**	**CCCTTCCTGTT**	**11**	**1.24E-03**

	ELF3	CCACTTCCTGGTTC	12	1.26E-03
	SP4	CCCGGCCCCGCCCCCTTCCC	19	1.33E-03
	ETV2	CCCACTTCCTGTTTCC	13	1.62E-03
	NKX25	CCTCTCCA	8	1.94E-03
	GABPA	CCACTTCCGGTTCC	12	2.39E-03
	**EGR1[*2]**	**CCCCCGCCCACGCCCTC**	**17**	**2.17E-05**
	PATZ1	CCTCCCCCCCCGCCCCCTCCCC	19	3.62E-05
	**SP1[*2]**	**CCCCCCCCCGGCCCCGCCCCCC**	**20**	**4.96E-05**
	SP3	CCCCGGCCCCGCCCCCCCCC	18	1.08E-04
	KLF1	CCCGGCCCCGCCCC	14	3.27E-04
**TF motif – II**	KLF15	CCCCCCCCTGCTCCTCCCC	19	4.10E-04
	WT1	CCCCCCCTCCTCCCCCGCCC	18	4.33E-04
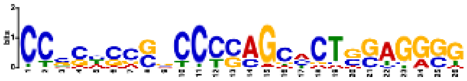	SP2	CCCCCGGCCCCGCCCCCCCCCC	19	7.52E-04
	KLF6	CCCCCGGCCCCGCCCTTCC	19	8.71E-04
	E2F6	CCCTTCCCGCCCC	13	1.02E-03
	ZN770	GATCCTCCCGCCTCAGCCTCCC	21	1.65E-03
	ZBTB6	CGGCTCCAGCACC	13	1.91E-03
	MAZ	CCCCCCCCCCCCCCCCCTCCCCC	20	2.13E-03
	ZN770	GATCCTCCCGCCTCAGCCTCCC	21	2.72E-11
	ZSC22	CCTCCTCCCTCAGACC	16	2.29E-04
	WT1	CCCCCCCTCCTCCCCCGCCC	20	5.61E-04
**TF motif – III**	ZN263	CTCCTCCTCTCCCTCCTCCC	20	5.61E-04
	**IKZF1[*2]**	**TCTCCCAA**	**8**	**7.62E-04**
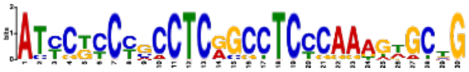	PATZ1	CCTCCCCCCCCGCCCCCTCCCC	22	1.40E-03
	KLF6	CCCCCGGCCCCGCCCTTCC	19	1.45E-03
	MAZ	CCCCCCCCCCCCCCCCCTCCCCC	22	1.84E-03
	ZN281	TCCCCTCCCCCACCC	15	2.01E-03

	ZN467	CCCCCCCCCCCCTCCCCTCCCC	22	2.10E-03

*1 The P-value (see **Supplementary Figure** 16, **Supplementary Figure** 20, **Supplementary Figure** 22) is the significance of know TF identified to the MEME identified TF motif.

*2 The red highlighted six known TFs (IKZF1, EGR1, SP1, ERG, ETS1, and ETV4) are the ones that also predicted as upstream regulators by IPA using gene expression data (**Supplementary Figure** 19, **Supplementary Figure** 20, **Supplementary Figure** 22).

Supplementary Table 5 – ***MEME discovered 7 novel TF motifs and their associated known TF motifs that were not supported by IPA predicted TFs.***

**MEME TF binding site motif**	**Known TF**	**Know TF binding site consensus**	**Overlap**	MEME Mixture Model Likelihood **p-value** *1
**TF motif – IV** 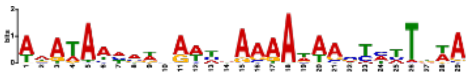	MEF2D	CTAAAAATAGCA	12	3.06E-04
	AIRE	TTAACCAATAAAACCAAT	18	3.41E-04
	PRDM6	AAAAAGAAAAAAA	13	7.98E-04
	MEF2C	GCTAAAAATAGCA	13	7.98E-04
	MEF2B	CCAAAAATAGCAAC	14	8.02E-04
	FOXJ3	AAAAAATAAACAA	13	1.15E-03
	ANDR	AGCAAACAAAAAAGAACA	18	1.43E-03
	NKX61	CATTAAATTCCCATTAATC	17	1.57E-03
	MEF2A	GCTAAAAATAGAA	13	2.03E-03
**TF motif – V** 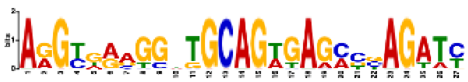	TBX3	GGAGGTGGGAA	11	1.04E-03
	MYB	GGTGGCAGTTGG	12	1.83E-03
	RARG	AGGTCAGAGTGACCTGGG	18	1.90E-03
	ETV5	GAGCAGGAAGTGAG	14	2.12E-03
	SMAD2	AGGTGACAGACA	12	2.40E-03
**TF motif – VI**	TWST1	ACATCTGGTTTTAATTA	15	1.18E-03

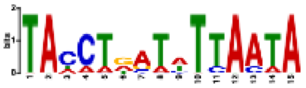				
**TF motif – VII**				
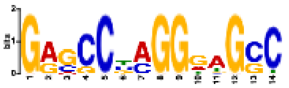	ZN320	GTGGGACCAGGGGGCCAGTG	14	1.66E-03
**TF motif – VIII**	ZN257	AGAGGCAAGAGC	12	5.47E-04
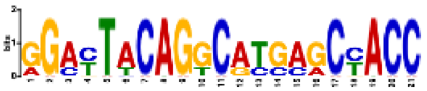	TEAD4	ACATTCCTGGCAT	13	2.49E-03
	SOX2	TCCCTTTGTTCTC	13	1.06E-04
**TF motif – IX**	PRDM6	TTTTTTTCTTTTT	13	7.26E-04
	ZN680	CCTCATTCTTCTTGGACATG	20	1.21E-03
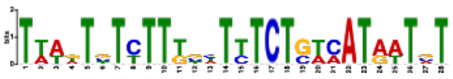	NFAC1	TTTCTTTTTTTCCAT	15	1.22E-03
	SOX4	TCCTTTGTTCTC	12	1.31E-03
	CDX2	TTTTATTGCTGT	12	1.99E-03
**TF motif – X**	ZN502	GAATGGAATCGAATGGAATC	14	1.65E-03
	ZN140	TAGGAGCGGAATTGCTGGGTCATA	14	1.80E-03
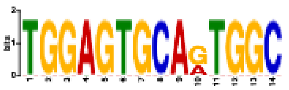	ZNF18	GGTGTGAACTGG	12	2.09E-03

*1 The P-value (see **Supplementary Figure** 16, **Supplementary Figure** 20, **Supplementary Figure** 22) is the significance of know TF identified to the MEME identified TF motif.

*2 The red highlighted six known TFs (IKZF1, EGR1, SP1, ERG, ETS1, and ETV4) are the ones that also predicted as upstream regulators by IPA using gene expression data (**Supplementary Figure** 19**, Supplementary Figure** 20**, Supplementary Figure** 22).

4 SUPPLEMENTAL METHODS

4.1 Generation of complete HMO biosynthesis network

Similar to Spahn et al (2016)[10], a generic reaction network for HMO biosynthesis is constructed based on a set of reaction rules (**Figure 3**A).

Based on the knowledge of enzyme specificities for each structure feature, the algorithm constructs the complete biosynthesis reaction network required to generate all HMOs of a user-defined complexity level. Specifically, the complexity level CL^k refers to the set of HMOs that can be produced from the initial lactose in *k* reaction steps (iterations) or less, following the reaction rules (**Figure 3**B).

The network generated is a complete network representing all reactions that, theoretically, could occur in human for a defined HMO structure complexity level, given the known enzyme specificities. We set the maximum complexity level to CL^{11}, as it captures the most complex HMO within the scope of this study (FDSLNH candidates, **Supplementary Figure** 5). In total, the network includes 9918 reactions and unique 2961 HMO structures.

4.2 Reduction of the generic network to a reduced network using Flux Variability approach

To make the network specific to the HMOs of interest in this study, the generic network is tailored by removing all the reactions that are not required to describe the synthesis of 16 measured HMOs. First, several additional reactions were introduced into the generic network to account for the structural ambiguity of uncharacterized HMOs (**Figure 3**B-C).

Second, the size of the generic network is reduced by removing all reactions that cannot contribute to the synthesis of the measured HMOs using Flux Variability Analysis[12] (**Figure 3**C, Supplemental Methods S5.4). The reduced generic network includes 221 reactions and 101 HMO structures (**Supplementary Figure** 10).

4.3 Generation of all minimal subnetworks for every potential HMO structure combination

From the reduced generic network, it is possible to enumerate all the subnetworks presenting specific combinations of candidate HMO structures for a defined number of reactions using MILP (S5.5). To this end, all potential combinations of the HMOs associated with unknown structures have first been generated (i.e. number of combinations equal to 3087). For each combination, we used a mixed integer linear programming algorithm to extract all subnetworks, which present unique biosynthetic pathways for every HMO (**Supplementary Figure** 6). Doing so, the number of reactions included in the subnetwork will range between the minimal number of reactions required for the simultaneous biosynthesis of all observed HMOs and the maximal number of reactions after which the extracted subnetwork no longer present unique solution when predicting the flux distribution. This methodology allowed the systematic

analysis of the entire solution space of the reduced generic network and identification of 44,984,988 existing alternative minimal subnetworks capable of synthesizing all measured HMOs.

4.4 Assessment of consistency of experimental data with subnetworks and selection of candidate models

We computed a normalized flux, i.e. the sum of the ratio between the flux of a reaction and its downstream reaction flux for every reaction associated to a specific linkage type within a subnetwork. For this, we first computed the flux values for every reaction within each subnetwork based on a measured glycoprofile in a milk sample, using Flux Balance Analysis[12] (FBA, *S5.4*). Since subnetwork extraction identifies the minimum number of reactions, no alternate optimal solutions were needed, thus resulting in a unique flux distribution for each subnetwork. We normalized the flux value (f) of a reaction by the value of flux associated with the previous reaction (f') within the subnetwork to obtain a ***flux ratio*** ($\underline{f}$) for each reaction.

$$\underline{f} = f/f'$$

Based on the assumption that only one enzyme catalyzes the formation of a linkage type, we compute the ***normalized flux*** ($\underline{f}_{l\omega D_m}$) as the sum of all flux ratios associated with a specific linkage (l). Since each flux ratio corresponds to a candidate model, ω, and parameterized on the glycomic data, D_m:

$$for\ a\ given\ \omega\ \&\ D_m, \qquad \underline{f_l} = \sum_{r \in l_r} \underline{f_r}$$

Doing so, every subnetwork is associated with 10 normalized flux values for each HMO measurement. The normalized flux values represent the activity levels of the 10 types of transferase reactions required for the biosynthesis of measured HMOs. We then computed the Spearman correlations (ρ) within linkage, l, between the flux ratio, $\underline{f}_{l\omega D_m}$, and the mRNA expression data ($E: g \rightarrow D_g$) of one of its associated candidate genes ($g \in l$, **Table 1**) for the subnetwork (ω). The ***gene-linkage score*** (ρ_{gl}) is defined as

$$for\ a\ given\ \omega, D_g, \&\ D_m, \qquad \rho_{gl} = cor(E(g_l) \sim \underline{f_l})$$

4.4.1 Computation of Subnetwork Score

The ***model score*** (S_ω) for each candidate model ω is defined as the average of the maximum z-score normalized gene-linkage score. The z-score, *z()*, mean and standard deviation is computed from the correlations of all gene-flux correlations within a linkage. The function z will denote this normalization from a set of correlations x to a normalized set of values relative to other correlations within that linkage $z_l: x \rightarrow N(x, \mu_l, \sigma_l)$.

$$for\ a\ given\ \omega, D_g, \&\ D_m, \qquad S_\omega = mean_{l \in L}\, \{\, z_l(max_{g\, \in\, l_g}\ \rho_{gl}\,)\}$$

The model score (S_ω) describes the average consistency between the most consistent genes and the associated fluxes in a subnetwork model.

4.4.2 Ranking subnetwork performance and extraction of candidate models

The distribution of subnetwork scores is approximately normal. Thus, we rely on a z-statistic to define the 95th percentile of high-performing models. Z-scores were calculated using the mean and standard deviation of the subnetwork scores for all subnetworks. Models with a z>1.646 were retained as high-performing models.

A careful reader may note that, though the total number of models does not change, the number of high-performing models is different between cohorts. This is because of small differences in skew. We used a z-score defined 95th percentile because the extrema were more robust to skew.

We also note that the ranking method is a z-score normalization. Because we used a z-score and a familiar critical z, it is easy to mistake this step for a statistical test; it is not. We did intend to select the top 5% of models as heuristically higher ranking, not as statistically "correct" (therefore, thresholding, cutoffs, or multiple test corrections are not relevant at this stage). Heuristic thresholding is a common and necessary practice in discretized ranking tests. This method will behave similarly to hypergeometric enrichment performed in differential expression; results are generally robust to reasonable changes in the p-value, fold-change, and base mean thresholds. Theoretically, robustness could be improved by avoiding the threshold assertion using a continuous over-representation method (e.g., Kolmogorov Smirnov test, the central innovation of GSEA). But computational constraints (>40 million models vs. 20 thousand genes), these methods were infeasible.

4.4.3 Summary network Extraction from the Reduced Network

For each reaction in the Reduced Network (**Figure** 3D) we measured the proportion of models in the top-performing model set which used each reaction (p^*), the proportion of models in the background model set which used each reaction (p), and the enrichment of each reaction in the top-performing model set compared to the background model set ($e(p^*|p)$ (**Supplementary Figure** 9).

The Summary network contains the most important paths in the reduced network where importance (i) is defined as $i = p^* + p^*e(p^*|p)$. This provides an additional reward for enrichment without inflicting a penalty on essential reactions which are not enriched in the top-performing model set. The important paths are defined as the top 5% of paths from lactose to an observed HMO where a path-score is defined as the sum of reaction importance between lactose and each observed HMO. The reduced network (**Supplementary Figure** 10) and Summary network (**Figure** 4) are presented with line thickness corresponding to importance.

4.5 Resolution of ambiguous genes and HMO structure through analysis of candidate models and subnetwork statistics

The top-performing candidate models from all subnetwork models were examined for distinguishing and common features that contributed to their extreme model score. To determine model features associated

with improved model performance, we compared model features of the high scoring candidate models to the background of all subnetwork models.

4.5.1 Enrichment of ambiguous HMO structures within top-performing candidate models

Towards disambiguating HMO structures enumerated in **Supplementary Figure** 5, we employed two strategies. First, we asked which structures were most enriched. Then, to further clarify ambiguous structures, co-inclusion of ambiguous structures was considered.

We first identified HMO structures that are more prevalent in the top-performing models, compared to all other models. We only consider structures that were included in >1% of all models. Hypergeometric enrichment was computed by comparing the occurrence of each structure in a top-performing set with its occurrence in the background. Those structures with both the highest prevalence in the subnetwork models and significant enrichment over the background were top-performing as the representative structure for that HMO. Those HMOs for which a single clear representative did not arise were examined for co-occurrence with sugars clearly resolved in the initial examination of prevalence and occurrence.

Co-occurrence network analyses involved selection of the top 10% of co-occurring structures; those structures most frequently co-occurring in top-performing models. Co-occurrence was measured by the Jaccard index (ratio of shared HMO structures over the union of HMO structures between cohort 1 and 2 with only the top-performing (10%) common models). The top 10% of Jaccard indexes were significant (hypergeometric enrichment p<<0.01). Structures with high centrality (alpha-centrality) were further highlighted (network edge weight was the exponentiated alpha centrality). Specifically, structures chosen in this analysis showed significant co-occurrence with at least three members of the central clade; the group containing DFLNT1/2 and DFLNH2.

4.5.2 Enrichment of ambiguous enzymes within top-performing candidate models

We identified genes potentially responsible for reactions listed in **Table** 1 as follows. Candidate model scores, S_{ω}, were calculated as the arithmetic mean of maximum gene-linkage scores, ρ_{gl} (S1.1.4). The maximum gene-linkage score will be contributed by the gene expression that best explains the flux predicted by a given model. To expand the model score to a gene-specific interrogation we employ three metrics.

The ***enrichment proportion*** (PROP) describes the increase in the proportion of high-performing models (relative to all models) that prefer a specific gene(**Supplementary Figure** 11); the proportion of models where a gene provides the maximum gene-linkage score within a model. It asks if that proportion is significantly higher in the top-performing models (Ω^* top 5% of model score) compared to the background models (Ω). We can ask the number of times a gene (γ) has the best correlation within a linkage for models in the top-performing (Ω^*) and background (Ω) sets:

$$B_{\gamma l} = \{\omega \in \Omega | argmax_{g \in l_g} \; \rho_{gl} \; = \gamma\} \qquad\qquad B^*_{\gamma l} = \{\omega \in \Omega^* | argmax_{g \in l_g} \; \rho_{gl} \; = \gamma\}$$

Then we can ask, for each gene, if $B^*_{\gamma l} \; > \; B_{\gamma l}$ using a hypergeometric enrichment:

$$HypGeo_{\gamma l} \; (k = \; |B^*_{\gamma l}| \; , n = |\Omega| \; \; , K \; = \; |B_{\gamma l}| \, , N \; = \; |\Omega| \;)$$

The enrichment measure helps to identify high performing genes by focusing only on the best performing gene in each model.

The ***gene-linkage score metric*** (GLS), described in section S1.1.4, is the Spearman correlation between gene expression and normalized flux: $\rho_{gl} = cor(E(g) \sim \underline{f}_{l_r\,\omega\,D_m})$. This metric provides information on every gene in every model regardless of the relevance of the gene to the model. This helps identify genes with low congruence because every gene is evaluated.

The ***model score contribution*** (MSC) is the Pearson correlation between the model score and the gene-linkage score for a specific gene, $cor(S_\omega \sim \rho_{gl})$. This is a more continuous approach to the question posed by the enrichment metric. This is a high granularity metric that can distinguish genes that highly influence the overall score.

5 THEORY, CALCULATIONS AND DEFINITIONS

5.1 Defining the Model-Space: Input date, Reaction Rules, Complete Network and Reduced Network

Our examination relies on two datatypes: glycomic data from High-Performance Liquid Chromatography (HPLC) and microarray data from Illumina Beadchip arrays. These datasets will be referred to as $D = \{D_m, D_g\}$ where D_m refers to the glycomic data an D_g refers to the gene expression data.

Let M_1, be a set of monosaccharides: $M_1 = \{Fuc, NeuAc, Glc, Gal, GlcNAc\}$. Let the set of multimeric sugars, M**,** be defined as all biochemically feasible HMOs up to a specified complexity; those which contain a maximum of k monosaccharides. Let R, be the set of all feasible reactions, specifically monosaccharide additions, resulting in an HMO in M. These reactions will be represented as an ordered set including a reactant (q), product (p), and monosaccharide (a).

$$R = \{r =< q, p, a > \in R \mid \forall_{p\in M} \exists_{q\in M} \exists_{a\in M_1}\ q + a \rightarrow p\ \}$$

The definition of HMOs (**M**) and reactions (**R**), allows us to define the complete HMO biosynthetic network $\Psi = \{M, R\}$, a directed acyclic graph rooted in a lactose (**Figure** 3B). Let Ω, the Reduced Network (**Figure** 3C-D), be a subgraph of the complete HMO biosynthetic network. We define the Reduced network as a subgraph of the Complete network (**Figure** 3B), $\Omega \subset \Psi = \{M, R\}\ s.t. M \subset M \wedge R \subset R$. The vertices of this network are taken from the set of HMOs, M, and the directed edges are taken from the set of reactions, R, such that $\Omega = \{M, R\}$. All HMOs which are retained in the reduced network, Ω, $m \in M$, m is an observed HMO ($m \in D_m$), or can be transformed into an observed HMO through a combination of reactions $r \in R$.

$$M = \{\ m \in M \mid \forall_{m\in M} m \in D_m \vee \exists_{\langle r_1,\ldots,r_n\rangle\, \in\, R^{[0,n]}}\ r_n\big(\ldots r_1(m)\big) \in D_m\ \}$$

For all reactions retained in the reduced network, Ω, $r \in R$, there exists an HMO which requires at least r to be successfully transformed into an HMO observed by HPLC ($m \in D_m$).

$$R = \{r \in R \mid \forall_{r\in R} \exists_{m\in M} \exists_{\langle s_1,\ldots,s_n\rangle \in R^{[0,n]}}\ s_n\big(\ldots s_1(m)\big) \wedge r \in \langle s_1, \ldots, s_n\rangle\}$$

This reduction of the complete network is done using Flux Variability Analysis.

5.2 Identifying Candidate Models within the Reduced Network

The Mixed Integer Linear Programing generated candidate models (**Figure** 3E), are subgraphs of the reduced network $\omega \in \Omega$. These subgraphs are sufficient to simulate the observed glycomic data with Flux Balance Analysis (FBA). Given an HMO dataset (D_m), where each quantity $d_{m_i} \in D_m$ defines an observed HMO (m). We refer to d_{m_i} as the quantification of HMO m_i and we refer to the flux through the reaction leading to m_i as f_{r_i}. We define flux as $f_{\omega,D_m} = FBA(\omega, D_m) = \langle f_{r_1}, \dots f_{r_n} \rangle_{\omega,D_m}$ (**Figure** 3F). To obtain the flux for a given reaction, r_i, we define a function $h: r_i \rightarrow f_{r_i}$. For a candidate model to be valid the flux distribution must match the data and be unique. Uniqueness means that no other flux distribution in the given model would reproduce the observed data. Matching the observed data means that the sum of flux through HMO-specific sink reactions (exiting/observed flux) is equivalent to the values observed in $m \in D_m$.

$$MILP(\Omega) = \{\omega = \{M', R'\} \subset \Omega \mid \forall_{d_{m_i} \in D} \exists_{f_{m_i} \in f_{\omega,D}} d_{m_i} = f_{m_i} \wedge \forall_{f_{m_i} \in f_{\omega,D}} f_{m_i} = f_{m_i} \}$$

5.3 Defining the Candidate Model Score

The Model Score (S) is defined by the best correlations between simulated flux and gene expression which may be supporting that flux. We first group similar reactions into 10 groups (**Table** 1) referred to as the linkages (L). The linkages are groups of reaction, $r \in R$, which involve equivalent monosaccharide additions of the same "type;" same monosaccharide added to the same terminal sugar via the same $\frac{\alpha}{\beta}$ configuration on the same carbons. Reactions of the same types are assumed to be supported by the same gene expressions. Therefore, each linkage $l \in L$ maps to both reactions of the same type and genes which may support those reactions: $l = \{ l_r, l_g \}$. Every reaction-linkage set $l_r \in l \in L$ is a set which contains several equivalent reactions $\forall_{l \in L} \forall_{r,s \in l} type(r) = type(s)$. All the linkage sets are disjoint as no reaction contains the addition of multiple monosaccharides,

$$\cup_{l_r \in l \in L} l_r = L \text{ but } \cap_{l_r \in l \in L} l_r = \emptyset$$

Each flux in this calculation is normalized to a proportion of the flux of its parent reaction $\underline{f} = \frac{f}{f'}$ where f' is the reaction which feeds f. Flux through a linkage is calculated as the sum of flux through all reactions within a linkage. Recall, every flux distribution, $f_{\omega,D_m} = FBA(\omega, D_m) = \langle f_{r_1}, \dots f_{r_n} \rangle_{\omega,D_m}$, is defined by the structure specified by a candidate model (ω) and parameterized by the glycomic data (D_m). Therefore, we can specify the linkage-flux $\underline{f}_{l_r \in l \in L}$ as:

$$\underline{f}_{l_r\,\omega\,D_m} = \sum_{r \in l_r} \frac{h(r)}{h(r)'} = \sum_{r \in l_r} \frac{f_r \in f_{\omega D_m}}{f_r' \in f_{\omega D_m}} = \sum_{r \in l_r} \underline{f}_{r\,\omega\,D_m}$$

Every gene-linkage set $l_g \in l \in L$ contains genes which are suspected to support the reactions in $l_r \in l$. The gene linkage sets are not disjoint because several linkages involve the same monosaccharide and further specification about the type of reaction the associated genes are expected to perform is not yet available. Therefore,

$$\cup_{l_g \in l \in L} l_g = L \text{ but } \cap_{l_g \in l \in L} l_g \neq \emptyset$$

The model score (S) for each candidate model ω can be defined as the average of the maximum normalized correlation between predicted flux through a given reaction and the gene expression predicted to support it. The normalization is a z-score normalization where all values are converted to z-scores using the mean (μ) and standard deviation (σ) of a common group. In this case, the z-score mean and standard deviation is computed from the correlations of all genes-flux correlations within a linkage. The function z will denote this normalization from a set of correlations x to a normalized set of values relative to other correlations within that linkage $z_l: x \to N(x, \mu_l, \sigma_l)$. We will also refer to the expression of a gene (g) as $E(g)$.

$$S_\omega = \left\{ z_l \left(cor \left(E(g) \sim f_{\underline{l}_r \; \omega \; D_m} \right) \right) \right\}$$

5.4 Flux Balance Analysis (FBA) and Flux Variability Analysis (FVA)

Flux Balance Analysis (FBA)[12] calculates a unique flux distribution (i.e., metabolic fluxes through all reactions in a network) using Linear Programming (LP) (i.e., minimization or maximization of one specific reaction of a network - equation 1) while Flux Variability Analysis[12] performs a min-max LP problem (i.e. each reaction of the network is maximized and subsequently minimized - equation 2). FBA calculates the flux vector that promotes a specific reaction within a network (described in equation 3) while satisfying a set of constraints (defined in equation 4). While the optimal value for a defined vi is always unique, the associated flux distribution v is usually not unique, as the LP problem is underdetermined. Thus, FVA can be used to evaluate the minimum and maximum range of each reaction flux that can still satisfy the systems of linear equations and associated constraints (equations 3-4)[12–14].

$$Min \; or \; Max \; v_i \qquad (1)$$

$$v_{i,lower} / \, v_{i,upper} = Min \, / \, Max \; v_i \qquad \forall v_i, i = 1, \ldots, N \qquad (2)$$

Subject to

$$Sv = 0 \qquad (3)$$

$$lb \leq v \leq ub \qquad (4)$$

where v is the vector of specific reaction rates (i.e. metabolic fluxes), N is the number of reaction fluxes in v , lb and ub are respectively the constraints set on the lower and upper bounds for the individual flux values, $v_{i,upper}$ and $v_{i,lower}$ are respectively the feasible upper and lower values of each flux v_i satisfying the system of linear equations (equations 3-4).

*FBA and FVA were used for two different step of the procedure of the Model Assembly (Figure 3). FVA was used t*o trim the Complete Network *into a reduced one containing only feasible biosynthetic reactions (Figure 3C). For this step,* the lower and upper bounds constraints of all reactions leading to the secretion of HMOs were set to zero except for the ones leading to the 16 experimentally-measured HMOs which were set to a value of 1/16 in order to account for their synthesis. *FBA was used further in the procedure to* compute the flux distribution necessary to synthesize the HMOs up to the measured concentrations using FBA*(Figure 3F). For this step,* the lower and upper constraint bounds (lb and ub) of the 16 sink

reactions corresponding to each measured HMOs were set to a value corresponding to the relative concentration in HMOs observed (concentration of the specific HMO divided by the total amount of HMOs). FBA is solved using the function *optimizeCbModel* and FVA using the function *fastFVA* both available in the CobraToolbox 3.0[15].

5.5 Mixed-Integer Linear Programming (MILP)

The enumeration of all alternate minimal reaction sets solving equally an LP such as $Sv = 0$ can be formulated with the following recursive MILP problem[108,109]:

$$Min\ Z = \sum_{i=1}^{N} w_i \quad (1)$$

Subject to

$$Sv = 0 \qquad (2)$$

$$\sum_{i \in NZ^{K-1}} y_i \geq 1 \qquad with\ y_i \in \{0,1\} \qquad (3)$$

$$\sum_{i \in NZ^k} w_i \leq \left|NZ^k\right| - 1 \qquad with\ w_i \in \{0,1\}\ and\ k = 1{:}K-1 \qquad (4)$$

$$w_i + y_i \leq 1 \qquad \forall i \qquad (5)$$

$$w_i.lb \leq v_i \leq w_i.ub \qquad \forall i \qquad (6)$$

Where S is the stoichiometric matrix, v is the vector of specific reaction rates (i.e. metabolic fluxes), N is the number of fluxes in v, y and w are vector of binary variables and lb and ub are respectively the lower and upper bounds for the individual flux values. The lower and upper bounds are respectively set to 0 and 1 for all the reactions in the network except for the 16 reactions corresponding to the secretion of the experimentally-measured HMOs which are constrained to a value of 1/16 in order to account for their synthesis.

The set of constraints (3-6) are used for changing the basis and identifying a new extreme point corresponding to one of the alternate optima. Specifically, at each iteration, K, at least one of the non-zero fluxes from the previous solution (NZ^{K-1}) must be set to zero, where the binary variable y_i is 1 if that flux is selected to be removed from the basis at iteration K (equation 3). The binary variable w_i is subsequently forced to zero if y_i is one (equation 5), and the upper and lower bounds for that particular flux are then constrained to zero (equation 6). Equation 4 ensures that alternate bases are not revisited by eliminating at least one non-zero variable found in previous iterations. Typically, the search algorithm stops when no other solution with the same objective function can be found in the MILP. In the context of this work, we slightly modified this end criterion in order to investigate all minimal networks: the search stops when the generated networks no longer present a unique flux distribution using FVA. The MILP problem formulated as above is solved using the function *solveCobraMILP* in the[15].

6 SUPPLEMENTARY RESULTS

6.1 Candidate gene filtering by expression and acceptor specificity

We initialized our candidate gene lists naively, including all members of a gene family corresponding to each linkage reaction (**Table** 1). Here, we query existing literature and expression data to narrow the initial list of gene family members to those relevant to HMO biosynthesis (**Supplementary Dataset 2**). Overall, a candidate gene was excluded from further consideration if it was not measured in the microarrays and that non-expression was confirmed by an independent RNA-Seq or if the gene was documented to perform an irrelevant reaction. If a gene is unmeasured in microarray and measured in RNA-Seq, it cannot be evaluated or ruled out. We further validated the RNA-Seq data by ranking it relative to expression in normal human tissues from GTEx[16]. If literature and expression data conflict (e.g. a gene performing a relevant reaction is not expressed), conflicts are resolved by the independent RNA-seq data; a relevant gene is not part of our system if it is not expressed. Please refer to **Supplementary Dataset 2** for details on expression metrics of glycogenes and gene-filtering decisions.

We first considered the likelihood a gene may perform the corresponding reaction considering the existing literature[17] and databases such as glycogene database[18], BRENDA[19], Uniprot[20], MetaCyc[21], KEGG[22]. Because the characterization of these genes with HMO acceptors is historically limited, we gave special attention to recent work in HMO chemosynthesis[2]. If a gene was used by Prudden et. al. to perform a specific reaction, we know it can perform the reaction. Similarly, if the linkage reaction has been demonstrated previously in HMO, lactoceramide derived, or a similar (GalNAc or Glc instead of GlcNAc) context, we retain that gene for further consideration. Genes were excluded from further examination if characterized to use a different donor including B4GAT1, formerly B3GNT1[23,24], and B3GALNT1, formerly B3GALT3[25,26]). Similarly, genes were excluded from further examination if they were known to synthesize a different linkage (e.g. FUT8 and the α-1,6 Fucose addition[27,28]) or use an unrelated acceptor (e.g. glycosaminoglycan (GAG) glycosyltransferases like B3GNT7[29–31], B3GALT6[32,33] and B4GALT7[34,35]. Through review of existing literature, we were able to rule out six glycosyltransferases reducing the candidate genes from 54 to 48.

We further examined microarray gene expression data from cohort 1 (GSE36936) and cohort 2 (GSE12669) to determine which of the candidate genes were expressed; we summarized expression to the third quartile (Q3) or 75th percentile to account for infrequent expression. The microarrays failed to measure the expression of 25 glycogenes (**Supplementary Dataset** 2; Sheet "Expression Measured"); 2 genes lacked probes, 11 genes showed no expression (Q3=0) in one microarray, 15 genes showed no expression (Q3=0) in both microarrays. Negligible expression was confirmed in an independent RNA-Seq study of the milk fat globules in lactating women (GSE45669[1]) and comparison to global expression distributions in GTEx[16]; genes unmeasured in the microarrays that did not meet the expression criteria--Q3 TPM>2 or (Q3 TPM<=2 and Q3 TPM > 50% of GTEx samples)--were excluded from further consideration. Of the 25 unmeasured glycogenes, 17 were confirmed unmeasured in the Lemay RNA-Seq relative to GTEx and excluded from further consideration while 8 genes could not be confirmed as non-expressed. 2 of the 8 unconfirmed non-expressed genes are known to perform irrelevant reactions--FUT8 and B3GNT7; the remaining 6 genes--B3GNT9, FUT5, FUT9, FUT10, B3GALT5, and ST6GALNAC3--cannot

be ruled out or evaluated in this study due to a lack of measurement. Unfortunately, 3 of the 6 questionably unmeasured genes--FUT5, FUT9 and B3GALT5--were demonstrated to synthesize these reactions in HMO[2]. Because low B3GALT5 expression was marginal and isolated to one microarray, we chose to include it in later analysis. 4 genes used in the Prudden experiment were successfully excluded due to low and RNA-Seq confirmed non-expression--FUT1, ST3GAL4, GCNT2B, and ST6GALNAC5. Non-expression of GCNT2B was confirmed with exon-level re-analysis of the Lemay RNA-seq data (STARv2.5.4b alignment to GRCh38); nearly all of the reads aligning to the three exons (1a, 1b, and 1c) definitive of isoforms A, B and C, aligned to exon 1a. Ultimately, 25 genes were retained for further analysis, 22 genes were excluded due to confirmed low expression or catalytic irrelevance, and 6 were designated removed from further analysis without being ruled out.

6.2 Generation of 44,984,988 candidate models representing HMO biosynthesis

To explore how human mammary gland epithelial cells synthesize HMOs, we generated candidate models based on a complete network describing all possible reactions leading to the synthesis of all possible HMO structures. HMO complexity was limited to 7 monosaccharides added to the starting lactose. The complete network was pruned using flux variability analysis, and reactions are removed if they are not used to synthesize the 16 most abundant HMOs. Using mixed integer linear programming, we enumerated all subnetworks that can accurately simulate the synthesis of observed oligosaccharides. The MILP produced 48,914,738 subnetworks. 3,929,750 of these subnetworks were unable to uniquely (upper and lower bound flux were not equal) simulate the HPLC data leaving 44,984,988 candidate models for further examination. Each candidate model retained 43-54 reactions, 0.4-.5% of the reactions found in the complete model and 19.5-24.4% of the reactions retained in the Reduced Network (**Supplementary Table 2**). These models covered all the possible combinations of HMO synthesis by the 10 known glycosyltransferase families that could describe the synthesis of the HMOs in this study.

6.3 Selection of best performing models

We computed the model score S_ω using the glycoprofiling and transcriptomic data from two independent cohorts. When sorted, these scores reveal a sigmoidal trend (**Supplementary Figure** 8A, **Supplementary Figure** 8F) suggesting an enrichment of high and low scoring models. Scores falling into the top 5% of density of the normal distribution of scores were ***top-performing*** (Ω^*, red) to represent high performing models (Described and justified in *Ranking subnetwork performance and selection of candidate models* in Methods). We found 2.66 million top-performing models using cohort 1, and 2.32 million top-performing models using cohort 2 (**Supplementary Table 2**). 241,589 models were common to the top-performing in cohort1 and cohort 2. Fewer top-performing models were expected from dataset 2 because it is a smaller dataset, and therefore its predictive power is limited; this does not present an issue as the primary purpose of dataset 2 is validation. As an initial validation, we mapped top-performing models from dataset2 (red) onto the sigmoid for dataset 1 (**Supplementary Figure** 8B) and did the same for top-performing models from dataset 1 (red, **Supplementary Figure** 8G). We note that high performing models in both datasets appear at the top of the sigmoid for the other dataset suggesting the selection is consistent. We also note that the intersection of models top-performing in analysis parameterized by dataset 1 and dataset 2 also appear at the top of both sigmoids (red, **Supplementary Figure** 8C, **Supplementary Figure** 8H).

Normality of the score distributions was also examined using Q-Q plots and simple distribution visualization. The candidate model scores for dataset 1, $S_{\omega}(D_1)$, are normally distributed as evidenced by the nearly linear Q-Q plot (**Supplementary Figure** 8D). The candidate model scores for dataset 2, $S_{\omega}(D_2)$, is less normally distributed as evidenced by the moderate deviation in the Q-Q plot (**Supplementary Figure** 8I). The deviation in the Q-Q plot for dataset 2 scores is expected as it is a smaller dataset therefore there is a moderate skew to lower scores due to limited power. Since dataset 2 is not being used for discovery, but rather validation, this moderate skew is manageable.

To address the potential over-representation of type-I HMOs in our models, we examined the distribution of type-I and type-II in tetra- and pentasaccharides with known structures. Across samples, the median abundance of type-II HMOs, LNnT, LNFPIII and LSTc were 3.33%, 0.041%, and 2.68% of total nmol/mL while type-I HMOs of the same size, LNT, LNFPI, LNFPII, and LSTc, was 15.3%, 9.39%, 7.45% and 0.45% respectively. This confirms the greater abundance of type-I HMOs compared with the type-II structures in the glycomic profiles (**Figure** 2C). This Summary Network thus provides orientation in this underspecified space.

7 SUPPLEMENTAL DISCUSSION

7.1 Validity of Selected Transcription Factors

To further validate the selected genes, we analyzed the promoters and gene expression patterns for common regulatory elements; if these genes are co-regulated, during lactation, they should enrich for common transcription regulatory elements (Figure 7, Table 2, Supplementary Figure 17-22

). Two studies implicated IKZF1 in regulating α-1,2 fucosylation through high-throughput analyses including type-1-diabetes GWAS[36] and text-mining TF prediction[37]. SP1, a TF already implicated in prolactin reception[38], has been known to transcriptionally regulate gene expression in several β4-galactosyltransferases including B4GALT4[39] and B4GALT5[40–42]. Consistent with the miRNA proxy theory[43], SP1 can also regulate α-1,3-fucosyltransferase, FUT4, for Lewis Antigen X biosynthesis [144] through miR-29b. The ETS gene superfamily encodes TFs (e.g., ETS1, ETV4 and ERG) that bind a purine-rich DNA sequence through the ETS domain[44], and ST3GAL1 contains several TFBS in its promoter region, including ETS1[45]. Together, our results of the discovered TFs and TFBS motifs are consistent with those reported for regulating GTs in similar biosynthesis reactions.

7.2 Leveraging HMO pathway resolution to clarify natural variation in HMO composition

With the newly reduced space of HMO biosynthetic pathways and knowledge of the enzymes and their regulation will enable mechanistic insights into the relationship of maternal genotype and infant development. HMO concentrations vary dramatically between mothers creating sometimes unpredictable health risks for newborns. Numerous factors influence HMO composition. These include genetic factors influencing blood type, secretor and Lewis status[46–51], diet[11], geography[11,52]. Variations in

HMO composition influence infant susceptibility to infection[53–56], impact risk of developing diseases such as necrotizing enterocolitis[38] and influence development[6,57–59] and contribute to childhood obesity[60,61]. To elucidate the mechanisms leading to the heterogeneity among women, and its connection to child health, the differences in HMO composition often need to be interpreted in the context of all the biosynthetic pathways.

7.3 Leveraging HMO pathway resolution to facilitate chemoezymatic synthesis

Finally, once essential HMOs are identified, the knowledge presented here on the HMO biosynthetic network can provide insights for the industrial production of HMO as a nutraceutical. Currently, commercially available formula is fortified with galactooligosaccharide (GOS), fructooligosaccharide (FOS) and 2'-fucosyllactose (2'-FL). These can be probiotic[44], modulate immune development[45], and reduce pathogenic susceptibility[62]. As larger health-relevant HMOs emerge[63], there is a need to scale up their production. Chemoenzymatic synthesis is feasible[2,64–67], but cell-based production can be more easily scaled. The growth of microbial HMO production is limited by knowledge of the biosynthetic system[68,69]. Small HMOs like 2’FL[70–72] and larger molecules like LNT have been produced in *E. coli*[73]. Increased knowledge of enzymes and reactions collected in this work will expand the toolbox for metabolic engineering by narrowing and prioritizing the list of relevant glycosyltransferases to HMO biosynthesis.

8 Supplemental References

1. Lemay, D. G. *et al.* RNA Sequencing of the Human Milk Fat Layer Transcriptome Reveals Distinct Gene Expression Profiles at Three Stages of Lactation. *PLOS ONE* **8**, e67531 (2013).
2. Prudden, A. R. *et al.* Synthesis of asymmetrical multiantennary human milk oligosaccharides. *Proc. Natl. Acad. Sci.* **114**, 6954–6959 (2017).
3. Kobata, A. Structures and application of oligosaccharides in human milk. *Proc. Jpn. Acad. Ser. B* **86**, 731–747 (2010).
4. Ninonuevo, M. R. *et al.* A Strategy for Annotating the Human Milk Glycome. *J. Agric. Food Chem.* **54**, 7471–7480 (2006).
5. Mantovani, V., Galeotti, F., Maccari, F. & Volpi, N. Recent advances on separation and characterization of human milk oligosaccharides. *ELECTROPHORESIS* **37**, 1514–1524 (2016).
6. Bode, L. The functional biology of human milk oligosaccharides. *Early Hum. Dev.* **91**, 619–622 (2015).
7. Bailey, T. L. & Elkan, C. The value of prior knowledge in discovering motifs with MEME. *Proc. Int. Conf. Intell. Syst. Mol. Biol.* **3**, 21–29 (1995).
8. Gupta, S., Stamatoyannopoulos, J. A., Bailey, T. L. & Noble, W. S. Quantifying similarity between motifs. *Genome Biol.* **8**, R24 (2007).
9. Bao, B. *et al.* Correcting for sparsity and interdependence in glycomics by accounting for glycan biosynthesis. *Nat. Commun.* **12**, 4988 (2021).
10. Spahn, P. N. *et al.* A Markov chain model for N-linked protein glycosylation – towards a low-parameter tool for model-driven glycoengineering. *Metab. Eng.* **33**, 52–66 (2016).
11. Azad, M. B. *et al.* Human Milk Oligosaccharide Concentrations Are Associated with Multiple Fixed and Modifiable Maternal Characteristics, Environmental Factors, and Feeding Practices. *J. Nutr.* **148**, 1733–1742 (2018).

12. Orth, J. D., Thiele, I. & Palsson, B. Ø. What is flux balance analysis? *Nat. Biotechnol.* **28**, 245–248 (2010).

13. Burgard, A. P., Vaidyaraman, S. & Maranas, C. D. Minimal Reaction Sets for Escherichia coli Metabolism under Different Growth Requirements and Uptake Environments. *Biotechnol. Prog.* **17**, 791–797 (2001).

14. Gudmundsson, S. & Thiele, I. Computationally efficient flux variability analysis. *BMC Bioinformatics* **11**, 489 (2010).

15. Heirendt, L. *et al.* Creation and analysis of biochemical constraint-based models using the COBRA Toolbox v.3.0. *Nat. Protoc.* **14**, 639 (2019).

16. Carithers, L. J. *et al.* A Novel Approach to High-Quality Postmortem Tissue Procurement: The GTEx Project. *Biopreservation Biobanking* **13**, 311–319 (2015).

17. Taniguchi, N., Honke, K. & Fukuda, M. *Handbook of Glycosyltransferases and Related Genes*. (Springer Science & Business Media, 2011).

18. Narimatsu, H. Construction of a human glycogene library and comprehensive functional analysis. *Glycoconj. J.* **21**, 17–24 (2004).

19. Schomburg, I. *et al.* The BRENDA enzyme information system–From a database to an expert system. *J. Biotechnol.* **261**, 194–206 (2017).

20. Magrane, M. & UniProt Consortium. UniProt Knowledgebase: a hub of integrated protein data. *Database J. Biol. Databases Curation* **2011**, bar009 (2011).

21. Caspi, R. *et al.* The MetaCyc database of metabolic pathways and enzymes and the BioCyc collection of Pathway/Genome Databases. *Nucleic Acids Res.* **42**, D459–D471 (2014).

22. Kanehisa, M., Furumichi, M., Tanabe, M., Sato, Y. & Morishima, K. KEGG: new perspectives on genomes, pathways, diseases and drugs. *Nucleic Acids Res.* **45**, D353–D361 (2017).

23. Praissman, J. L. *et al.* B4GAT1 is the priming enzyme for the LARGE-dependent functional glycosylation of α-dystroglycan. *eLife* **3**, e03943 (2014).

24. Willer, T. *et al.* The glucuronyltransferase B4GAT1 is required for initiation of LARGE-mediated α-dystroglycan functional glycosylation. *eLife* **3**, e03941 (2014).

25. Amado, M. *et al.* A Family of Human β3-Galactosyltransferases: CHARACTERIZATION OF FOUR MEMBERS OF A UDP-GALACTOSE:β-N-ACETYL-GLUCOSAMINE/β-NACETYL-GALACTOSAMINE β-1,3-GALACTOSYLTRANSFERASE FAMILY *. *J. Biol. Chem.* **273**, 12770–12778 (1998).

26. Okajima, T. *et al.* Expression Cloning of Human Globoside Synthase cDNAs: IDENTIFICATION OF β3Gal-T3 AS UDP-N-ACETYLGALACTOSAMINE:GLOBOTRIAOSYLCERAMIDE β1,3-N-ACETYLGALACTOSAMINYLTRANSFERASE *. *J. Biol. Chem.* **275**, 40498–40503 (2000).

27. Yanagidani, S. *et al.* Purification and cDNA Cloning of GDP-L-Fuc:N-Acetyl-β-D-Glucosaminide:α1-6 Fucosyltransferase (α1-6 FucT) from Human Gastric Cancer MKN45 Cells1. *J. Biochem. (Tokyo)* **121**, 626–632 (1997).

28. Uozumi, N. *et al.* Purification and cDNA Cloning of Porcine Brain GDP-L-Fuc:N-Acetyl-β-D-Glucosaminide α1→6Fucosyltransferase *. *J. Biol. Chem.* **271**, 27810–27817 (1996).

29. Kataoka, K. & Huh, N. A novel β1,3-N-acetylglucosaminyltransferase involved in invasion of cancer cells as assayed in vitro. *Biochem. Biophys. Res. Commun.* **294**, 843–848 (2002).

30. Kitayama, K., Hayashida, Y., Nishida, K. & Akama, T. O. Enzymes Responsible for Synthesis of Corneal Keratan Sulfate Glycosaminoglycans *. *J. Biol. Chem.* **282**, 30085–30096 (2007).

31. Seko, A. & Yamashita, K. β1,3-N-Acetylglucosaminyltransferase-7 (β3Gn-T7) acts efficiently on keratan sulfate-related glycans. *FEBS Lett.* **556**, 216–220 (2004).

32. Bai, X. *et al.* Biosynthesis of the Linkage Region of Glycosaminoglycans: CLONING AND ACTIVITY OF GALACTOSYLTRANSFERASE II, THE SIXTH MEMBER OF THE β1,3-GALACTOSYLTRANSFERASE FAMILY (β3GalT6) *. *J. Biol. Chem.* **276**, 48189–48195 (2001).

33. Ju, T., Brewer, K., D'Souza, A., Cummings, R. D. & Canfield, W. M. Cloning and Expression of Human Core 1 β1,3-Galactosyltransferase *. *J. Biol. Chem.* **277**, 178–186 (2002).

34. Almeida, R. *et al.* Cloning and Expression of a Proteoglycan UDP-Galactose:β-Xylose β1,4-Galactosyltransferase I: A SEVENTH MEMBER OF THE HUMAN β4-GALACTOSYLTRANSFERASE GENE FAMILY *. *J. Biol. Chem.* **274**, 26165–26171 (1999).

35. Okajima, T., Yoshida, K., Kondo, T. & Furukawa, K. Human Homolog of Caenorhabditis elegans sqv-3 Gene Is Galactosyltransferase I Involved in the Biosynthesis of the Glycosaminoglycan-Protein Linkage Region of Proteoglycans *. *J. Biol. Chem.* **274**, 22915–22918 (1999).

36. Inshaw, J. R. J., Cutler, A. J., Burren, O. S., Stefana, M. I. & Todd, J. A. Approaches and advances in the genetic causes of autoimmune disease and their implications. *Nat. Immunol.* **19**, 674–684 (2018).

37. Rouillard, A. D. *et al.* The harmonizome: a collection of processed datasets gathered to serve and mine knowledge about genes and proteins. *Database* **2016**, (2016).

38. Hu, Z.-Z., Zhuang, L., Meng, J. & Dufau, M. L. Transcriptional Regulation of the Generic Promoter III of the Rat Prolactin Receptor Gene by C/EBPβ and Sp1 *. *J. Biol. Chem.* **273**, 26225–26235 (1998).

39. Sugiyama, A., Fukushima, N. & Sato, T. Transcriptional mechanism of the β4-galactosyltransferase 4 gene in SW480 human colon cancer cell line. *Biol. Pharm. Bull.* **advpub**, (2017).

40. Sato, T. & Furukawa, K. Transcriptional Regulation of the Human β-1,4-Galactosyltransferase V Gene in Cancer Cells: ESSENTIAL ROLE OF TRANSCRIPTION FACTOR Sp1 *. *J. Biol. Chem.* **279**, 39574–39583 (2004).

41. Sato, T. & Furukawa, K. Sequential Action of Ets-1 and Sp1 in the Activation of the Human β-1,4-Galactosyltransferase V Gene Involved in Abnormal Glycosylation Characteristic of Cancer Cells *. *J. Biol. Chem.* **282**, 27702–27712 (2007).

42. Zhou, L., Jiang, J. & Gu, J. β1,4-Galactosyltransferase: Regulation and Signaling in Cancers. in *Glycoscience: Biology and Medicine* (eds. Taniguchi, N., Endo, T., Hart, G. W., Seeberger, P. H. & Wong, C.-H.) 1141–1148 (Springer Japan, 2015). doi:10.1007/978-4-431-54841-6_74.

43. Kurcon, T. *et al.* miRNA proxy approach reveals hidden functions of glycosylation. *Proc. Natl. Acad. Sci.* **112**, 7327–7332 (2015).

44. Dhordain, P., Dewitte, F., Desbiens, X., Stehelin, D. & Duterque-Coquillaud, M. Mesodermal expression of the chicken erg gene associated with precartilaginous condensation and cartilage differentiation. *Mech. Dev.* **50**, 17–28 (1995).

45. Taniguchi, A., Yoshikawa, Itaru & Matsumoto, K. Genomic structure and transcriptional regulation of human Galβ1,3GalNAc α2,3-sialyltransferase (hST3Gal I) gene. *Glycobiology* **11**, 241–247 (2001).

46. Viverge, D., Grimmonprez, L., Cassanas, G., Bardet, L. & Solere, M. Discriminant carbohydrate components of human milk according to donor secretor types. *J. Pediatr. Gastroenterol. Nutr.* **11**, 365–370 (1990).

47. Kumazaki, T. & Yoshida, A. Biochemical evidence that secretor gene, Se, is a structural gene encoding a specific fucosyltransferase. *Proc. Natl. Acad. Sci.* **81**, 4193–4197 (1984).

48. Nishihara, S. *et al.* Molecular genetic analysis of the human Lewis histo-blood group system. *J. Biol. Chem.* **269**, 29271–29278 (1994).

49. Kudo, T. *et al.* Molecular Genetic Analysis of the Human Lewis Histo-blood Group System: II. SECRETOR GENE INACTIVATION BY A NOVEL SINGLE MISSENSE MUTATION A385T IN JAPANESE NONSECRETOR INDIVIDUALS (∗). *J. Biol. Chem.* **271**, 9830–9837 (1996).

50. Koda, Y., Soejima, M., Liu, Y. & Kimura, H. Molecular basis for secretor type alpha(1,2)-fucosyltransferase gene deficiency in a Japanese population: a fusion gene generated by unequal crossover responsible for the enzyme deficiency. *Am. J. Hum. Genet.* **59**, 343–350 (1996).

51. Thurl, S., Henker, J., Siegel, M., Tovar, K. & Sawatzki, G. Detection of four human milk groups with respect to Lewis blood group dependent oligosaccharides. *Glycoconj. J.* **14**, 795–799 (1997).

52. McGuire, M. K. *et al.* What's normal? Oligosaccharide concentrations and profiles in milk produced by healthy women vary geographically12. *Am. J. Clin. Nutr.* **105**, 1086–1100 (2017).

53. Morrow, A. L. *et al.* Fucosyltransferase 2 Non-Secretor and Low Secretor Status Predicts Severe Outcomes in Premature Infants. *J. Pediatr.* **158**, 745–751 (2011).

54. Morrow, A. L. *et al.* Human Milk Oligosaccharide Blood Group Epitopes and Innate Immune Protection against Campylobacter and Calicivirus Diarrhea in Breastfed Infants. in *Protecting Infants through Human Milk* (eds. Pickering, L. K., Morrow, A. L., Ruiz-Palacios, G. M. & Schanler, R. J.) 443–446 (Springer US, 2004). doi:10.1007/978-1-4757-4242-8_61.

55. Vandenplas, Y. *et al.* Human Milk Oligosaccharides: 2′-Fucosyllactose (2′-FL) and Lacto-N-Neotetraose (LNnT) in Infant Formula. *Nutrients* **10**, 1161 (2018).

56. Yu, Z.-T., Nanthakumar, N. N. & Newburg, D. S. The Human Milk Oligosaccharide 2′-Fucosyllactose Quenches Campylobacter jejuni–Induced Inflammation in Human Epithelial Cells HEp-2 and HT-29 and in Mouse Intestinal Mucosa. *J. Nutr.* **146**, 1980–1990 (2016).

57. Edmond, K. M. *et al.* Delayed Breastfeeding Initiation Increases Risk of Neonatal Mortality. *Pediatrics* **117**, e380–e386 (2006).

58. Bode, L. Human milk oligosaccharides: Every baby needs a sugar mama. *Glycobiology* **22**, 1147–1162 (2012).

59. Picciano, M. F. Nutrient Composition of Human Milk. *Pediatr. Clin. North Am.* **48**, 53–67 (2001).

60. Alderete, T. L. *et al.* Associations between human milk oligosaccharides and infant body composition in the first 6 mo of life. *Am. J. Clin. Nutr.* **102**, 1381–1388 (2015).

61. Uwaezuoke, S. N., Eneh, C. I. & Ndu, I. K. Relationship Between Exclusive Breastfeeding and Lower Risk of Childhood Obesity: A Narrative Review of Published Evidence. *Clin. Med. Insights Pediatr.* **11**, 1179556517690196 (2017).

62. Vos, A. P. *et al.* A specific prebiotic oligosaccharide mixture stimulates delayed-type hypersensitivity in a murine influenza vaccination model. *Int. Immunopharmacol.* **6**, 1277–1286 (2006).

63. Autran, C. A. *et al.* Human milk oligosaccharide composition predicts risk of necrotising enterocolitis in preterm infants. *Gut* **67**, 1064–1070 (2018).

64. Furuike, T., Yamada, K., Ohta, T., Monde, K. & Nishimura, S.-I. An efficient synthesis of a biantennary sialooligosaccharide analog using a 1,6-anhydro-β-lactose derivative as a key synthetic block. *Tetrahedron* **59**, 5105–5113 (2003).

65. J. Fair, R., Sik Hahm, H. & H. Seeberger, P. Combination of automated solid-phase and enzymatic oligosaccharide synthesis provides access to α(2,3)-sialylated glycans. *Chem. Commun.* **51**, 6183–6185 (2015).

66. Yao, W., Yan, J., Chen, X., Wang, F. & Cao, H. Chemoenzymatic synthesis of lacto-N-tetrasaccharide and sialyl lacto-N-tetrasaccharides. *Carbohydr. Res.* **401**, 5–10 (2015).

67. Prudden, A. R., Chinoy, Z. S., Wolfert, M. A. & Boons, G.-J. A multifunctional anomeric linker for the chemoenzymatic synthesis of complex oligosaccharides. *Chem. Commun.* **50**, 7132–7135 (2014).

68. Bode, L. *et al.* Overcoming the limited availability of human milk oligosaccharides: challenges and opportunities for research and application. *Nutr. Rev.* **74**, 635–644 (2016).

69. Guan, N. & Chen, R. Recent Technology Development for the Biosynthesis of Human Milk Oligosaccharide. *Recent Pat. Biotechnol.* **12**, 92–100 (2018).

70. Lee, W.-H. *et al.* Whole cell biosynthesis of a functional oligosaccharide, 2′-fucosyllactose, using engineered Escherichia coli. *Microb. Cell Factories* **11**, 48 (2012).

71. Chin, Y.-W., Kim, J.-Y., Lee, W.-H. & Seo, J.-H. Enhanced production of 2′-fucosyllactose in engineered Escherichia coli BL21star(DE3) by modulation of lactose metabolism and fucosyltransferase. *J. Biotechnol.* **210**, 107–115 (2015).

72. Baumgärtner, F., Seitz, L., Sprenger, G. A. & Albermann, C. Construction of Escherichia coli strains with chromosomally integrated expression cassettes for the synthesis of 2′-fucosyllactose. *Microb. Cell Factories* **12**, 40 (2013).

73. Baumgärtner, F., Conrad, J., Sprenger, G. A. & Albermann, C. Synthesis of the Human Milk Oligosaccharide Lacto-N-Tetraose in Metabolically Engineered, Plasmid-Free E. coli. *ChemBioChem* **15**, 1896–1900 (2014).
