# Supplementary material for: Elucidating Human Milk Oligosaccharide biosynthetic genes through network-based multi-omics integration
Source: Nat Commun. 2022 May 4;13:2455. doi: 10.1038/s41467-022-29867-4 (PMC9068700; doi:10.1038/s41467-022-29867-4)
Supplement: Supplementary file 2 — Reporting Summary [file 41467_2022_29867_MOESM2_ESM.pdf]

nature research

Corresponding author(s): Nathan E. Lewis

Last updated by author(s): Feb 8, 2022

Reporting Summary

Nature Research wishes to improve the reproducibility of the work that we publish. This form provides structure for consistency and transparency in reporting. For further information on Nature Research policies, see our Editorial Policies and the Editorial Policy Checklist.

Statistics

For all statistical analyses, confirm that the following items are present in the figure legend, table legend, main text, or Methods section.

n/a	Confirmed	
☐	☒	The exact sample size (n) for each experimental group/condition, given as a discrete number and unit of measurement
☐	☒	A statement on whether measurements were taken from distinct samples or whether the same sample was measured repeatedly
☒	☐	The statistical test(s) used AND whether they are one- or two-sided<br>*Only common tests should be described solely by name; describe more complex techniques in the Methods section.*
☐	☒	A description of all covariates tested
☐	☒	A description of any assumptions or corrections, such as tests of normality and adjustment for multiple comparisons
☐	☒	A full description of the statistical parameters including central tendency (e.g. means) or other basic estimates (e.g. regression coefficient) AND variation (e.g. standard deviation) or associated estimates of uncertainty (e.g. confidence intervals)
☐	☒	For null hypothesis testing, the test statistic (e.g. F, t, r) with confidence intervals, effect sizes, degrees of freedom and P value noted<br>*Give P values as exact values whenever suitable.*
☒	☐	For Bayesian analysis, information on the choice of priors and Markov chain Monte Carlo settings
☐	☒	For hierarchical and complex designs, identification of the appropriate level for tests and full reporting of outcomes
☐	☒	Estimates of effect sizes (e.g. Cohen's d, Pearson's r), indicating how they were calculated

Our web collection on statistics for biologists contains articles on many of the points above.

Software and code

Policy information about availability of computer code

Data collection	HPLC quantification of HMO data used Chromeleon 7.2.
Data analysis	Modeling of HMO biosynthesis was performed in Matlab 2016b using the CobraToolbox v3. All analysis of biosynthetic models, interpretation and statistics were performed in R v3.6. In R, we used bigmemory v4.5.36, bigalgebra v1.0.1 and biganalytics v1.1.21 to handle the millions of models and associated statistics. We used metap for pooling p-values. Custom code for the flux expression comparison is provided at https://github.com/bkellman/HMO_GeneReaction_pred

For manuscripts utilizing custom algorithms or software that are central to the research but not yet described in published literature, software must be made available to editors and reviewers. We strongly encourage code deposition in a community repository (e.g. GitHub). See the Nature Research guidelines for submitting code & software for further information.

Data

Policy information about availability of data

All manuscripts must include a data availability statement. This statement should provide the following information, where applicable:

- Accession codes, unique identifiers, or web links for publicly available datasets
- A list of figures that have associated raw data
- A description of any restrictions on data availability

We provide the HMO and gene expression data and MIRAGE metadata at https://github.com/bkellman/HMO_GeneReaction_pred/data/data_raw/. The expression datasets were previously published under GEO accessions: GSE36936 (cohort 1) and GSE12669 (cohort 2).

Field-specific reporting

Please select the one below that is the best fit for your research. If you are not sure, read the appropriate sections before making your selection.

- [x] Life sciences
- [ ] Behavioural & social sciences
- [ ] Ecological, evolutionary & environmental sciences

For a reference copy of the document with all sections, see nature.com/documents/nr-reporting-summary-flat.pdf

Life sciences study design

All studies must disclose on these points even when the disclosure is negative.

Sample size	Cohort 1 HMO data contains 48 samples from 6 mothers. Cohort 2 HMO data contains 10 samples from 5 mothers. Sampling was temporal between 1 and 42 days postpartum (DPP). Because these data were already collected, no prospective sample size calculations were performed. All statistical analyses and subsequent conclusions are cognisent of sample size therefore our conclusions are measured and appropriate given the sample size. Both cohorts were assembled, sampled, and published prior to this study. Sample size was not chosen for this study. Sample size was based on available data.
Data exclusions	HPLC failed to quantify HMO in the day 1 sample collected from subject L6, therefore, no data from this sample could be included.
Replication	Results were verified against well known phenomena in glycobiology. Regarding HMO measurements, absolute abundance of HMO is determined by a well-characterized low-noise method using HPLC analysis as previously described by McGuire et al (cite https://www.ncbi.nlm.nih.gov/pmc/articles/PMC5402033/) therefore, no technical replicates were performed.
Randomization	Samples were analyzed in a random order to avoid batch effects.
Blinding	All samples were blinded with respect to secretor status to technicians and researchers until the statistical analysis began. Investigators were not blinded to subject identity or secretor status due to irrelevance and infeasibility. Secretor status is obvious in any measurement and subject identity is not related to the final results.

Reporting for specific materials, systems and methods

We require information from authors about some types of materials, experimental systems and methods used in many studies. Here, indicate whether each material, system or method listed is relevant to your study. If you are not sure if a list item applies to your research, read the appropriate section before selecting a response.

Materials & experimental systems

n/a	Involved in the study	
x		Antibodies
x		Eukaryotic cell lines
x		Palaeontology and archaeology
x		Animals and other organisms
	x	Human research participants
x		Clinical data
x		Dual use research of concern

Methods

n/a	Involved in the study	
x		ChIP-seq
x		Flow cytometry
x		MRI-based neuroimaging

Human research participants

Policy information about studies involving human research participants

Population characteristics	Lactating women 18-35 years of age with uncomplicated singleton pregnancy, vaginal delivery at term (>37 weeks), Body Mass Index <26 kg/m2 without diabetes, impaired glucose tolerance, anemia, or renal or hepatic dysfunction were given informed consent before sample collection. Full description of characteristic in previous cohort publications (Mohammad, 2012; Maningat, 2009)
Recruitment	Subjects were recruited through an IRB-approved advertisement process. The study (and related studies) were advertised using in hospitals/websites/church/schools/shops/community centers and some other location frequented by new mothers or pregnant women. Interested women contacted the study organizers directly. All women who fulfilled the inclusion/exclusion criteria were engaged in-person by the research coordinator, research staff, or research investigator. The primary investigator or a designee followed the procedure for obtaining and documenting assent for the infant participants and consent from their mothers. After a detailed explanation, the potential participant had the opportunity of asking questions. Participants were free to withdraw the consent at any point and any time throughout the study process.
Ethics oversight	Samples were collected following Institutional Review Board approval (Baylor College of Medicine, Houston, TX)

Note that full information on the approval of the study protocol must also be provided in the manuscript.
