# Supplementary material for: Elucidating Human Milk Oligosaccharide biosynthetic genes through network-based multi-omics integration
Source: Nat Commun. 2022 May 4;13:2455. doi: 10.1038/s41467-022-29867-4 (PMC9068700; doi:10.1038/s41467-022-29867-4)
Supplement: Supplementary file 4 — Description of Additional Supplementary Files [file 41467_2022_29867_MOESM4_ESM.pdf]

Title: Supplementary Dataset 1 –
Description: HPLC and microarray data used for modeling. See methods for complete description of each dataset and cohort

Title: Supplementary Dataset 2 –
Description: Linkages and genes considered throughout this analysis. List of glycosyltransferase genes associated with each reaction and associated expression in datasets within and beyond this work. Here we list the 56 candidate glycosyltransferase genes considered. Columns **A-E** indicate the corresponding reaction, gene name and various identifiers. Column **G**, "Prudden," indicates if the gene was used Prudden et. al. 2017. Columns **H-J** show expression on cohort 1 and 2 microarrays (relative abundance) and an independent RNA-seq in the same tissue (TPM). Column **M-O** specify the distribution of TPMs in GTEx (min, Q1, Q2, Q3, Q4, max), the percentile where Q3 from the RNA-seq falls in the GTEx distribution, and the log2 Fold Change of RNA-seq Q3 vs GTEx Q3. Column **P** specifies any low expression events. Columns **Q-S** compare microarray and RNA-seq expression and usage in Prudden et. al. 2017. Column **T** indicates if previous literature found a gene relevant ("yes"), partially relevant ("similar") or irrelevant ("no") to the reaction in question; elaborated in Supplementary Dataset 2. Columns **U-V** delimited and justify which genes will be included for further analysis. The remailing columns provide some relevant detail on substrate specificity for each gene.

Title: Supplementary Dataset 3 -
Description: The FVA reduced HMO biosynthetic network including HMO and reaction calculation referenced throughout the manuscript
